# Humans strategically avoid connecting to others who agree and avert the emergence of network polarization in a coordination task

**DOI:** 10.1038/s41598-023-38353-w

**Published:** 2023-07-12

**Authors:** Nico Gradwohl, Ariana Strandburg-Peshkin, Helge Giese

**Affiliations:** 1 Department of Psychology, University of Konstanz, Konstanz, Germany; 2 Biology Department, University of Konstanz, Konstanz, Germany; 3 Centre for the Advanced Study of Collective Behaviour, University of Konstanz, Konstanz, Germany; 4 Department for the Ecology of Animal Societies, Max Planck Institute of Animal Behavior, Konstanz, Germany; 5 Charité-Universitätsmedizin Berlin, Berlin, Germany

**Keywords:** Psychology, Human behaviour, Behavioural methods

## Abstract

Clusters of like-minded individuals can impede consensus in group decision-making. We implemented an online color coordination task to investigate whether control over communication links creates clusters impeding group consensus. In 244 6-member networks, individuals were incentivized to reach a consensus by agreeing on a color, but had conflicting incentives for which color to choose. We varied (1) if communication links were static, changed randomly over time, or were player-controlled; (2) whether links determined who was observed or addressed; and (3) whether a majority existed or equally many individuals preferred each color. We found that individuals preferentially selected links to previously unobserved and disagreeing others, avoiding links with agreeing others. This prevented cluster formation, sped up consensus formation rather than impeding it, and increased the probability that the group agreed on the majority incentive. Overall, participants with a consensus goal avoided clusters by applying strategies that resolved uncertainty about others.

## Introduction

Social groups must often come to consensus on a single course of action despite differing preferences. Often, individuals only communicate with a subset of other group members^1^. The resulting communication networks can help or hinder the successful spread of information about others’ behaviors or opinions^2–4^. One frequent impediment to consensus formation is clusters of like-minded individuals^5,6^ or “echo chambers”^7,8^, which promote information exchange among individuals who already agree. Such clusters have been implicated as responsible for increased opinion polarization^9–11^, although their impact has also been called into question^12,13^. This study addresses the question of whether such opinion clusters already emerge from the way in which individuals with different preferences for the groups’ consensus select their communication links.

The outcome and speed of consensus formation is strongly affected by network structure^5,11^, the presence of conflicting opinions or preferences^4,14,15^, and the position of opinionated or incentivized individuals within the network^6^. For instance, imbalances in the connections determining observations can bias the consensus outcome by creating the false impression of a majority^11^ and can even make a minority seem to outnumber the majority^14^, creating a “majority illusion”^16^. Moreover, although clustered links can be beneficial to behavior spread^2^, they can also impede consensus formation^5^. If different opinions dominate in different areas of a clustered network, individuals interacting only with local neighbors may not encounter opposing opinions, resulting in deadlock.

In many situations, individuals have some control over the network structures that determine their access to information. Control over network links can be beneficial, since any dynamics, including random link updating, can help individuals to avoid clusters^17^. Moreover, link updating can help to create networks that are better at spreading information^18^ and connecting to the most competent others can aid the wisdom of the crowds^19^. This suggests that groups can benefit from individuals choosing to exchange information with specific others. However, individuals generally show a tendency for homophily, i.e., associating with similar others^20^. They break links to others who strongly disagree, leading to the formation of polarized clusters in theoretical models of opinion formation^21^ and Twitter networks^22^. The tendency to cluster is also apparent in dyadic interactions on networks where clusters of cooperators help to maintain cooperation^23–26^ and facilitate bilateral coordination^27^. Thus, even though control over network links has the potential to help consensus formation by avoiding clusters and promoting successful information spread, individuals’ persistent tendency to form homophilous clusters could also hinder consensus formation.

The direction of links is another aspect of communication network structure that likely affects consensus outcomes, but has rarely been addressed systematically. Opinion formation models^28,29^ suggest that the success of link-selection strategies can depend on the direction of links: when links determine who can be influenced, a preference for links to others who agree through breaking links with others who disagree, slows down consensus. Conversely, a preference for links to agreeing others speeds up consensus formation when links determine who can be copied. Crucially, group-wide bias towards a minority or towards one of equally-sized subgroups in local observations^11^ can only be observed when links are directed. To address the role of link direction for consensus formation, we conducted a set of simulations (see Supplementary Sect. 1 and materials in the pre-registration), suggesting that individuals can create a majority illusion to their disadvantage as a function of link direction: (a) when they have control over their outgoing links (determining who sees them), and send to others who agree, or, (b) conversely, when they have control over their incoming links (determining their observations), and observe others who disagree. Thus, individuals should be sensitive to link direction. They should avoid assortment more when they determine who observes them, but seek out assortment to reinforce their choice when links determine who they observe.

**Figure 1:**
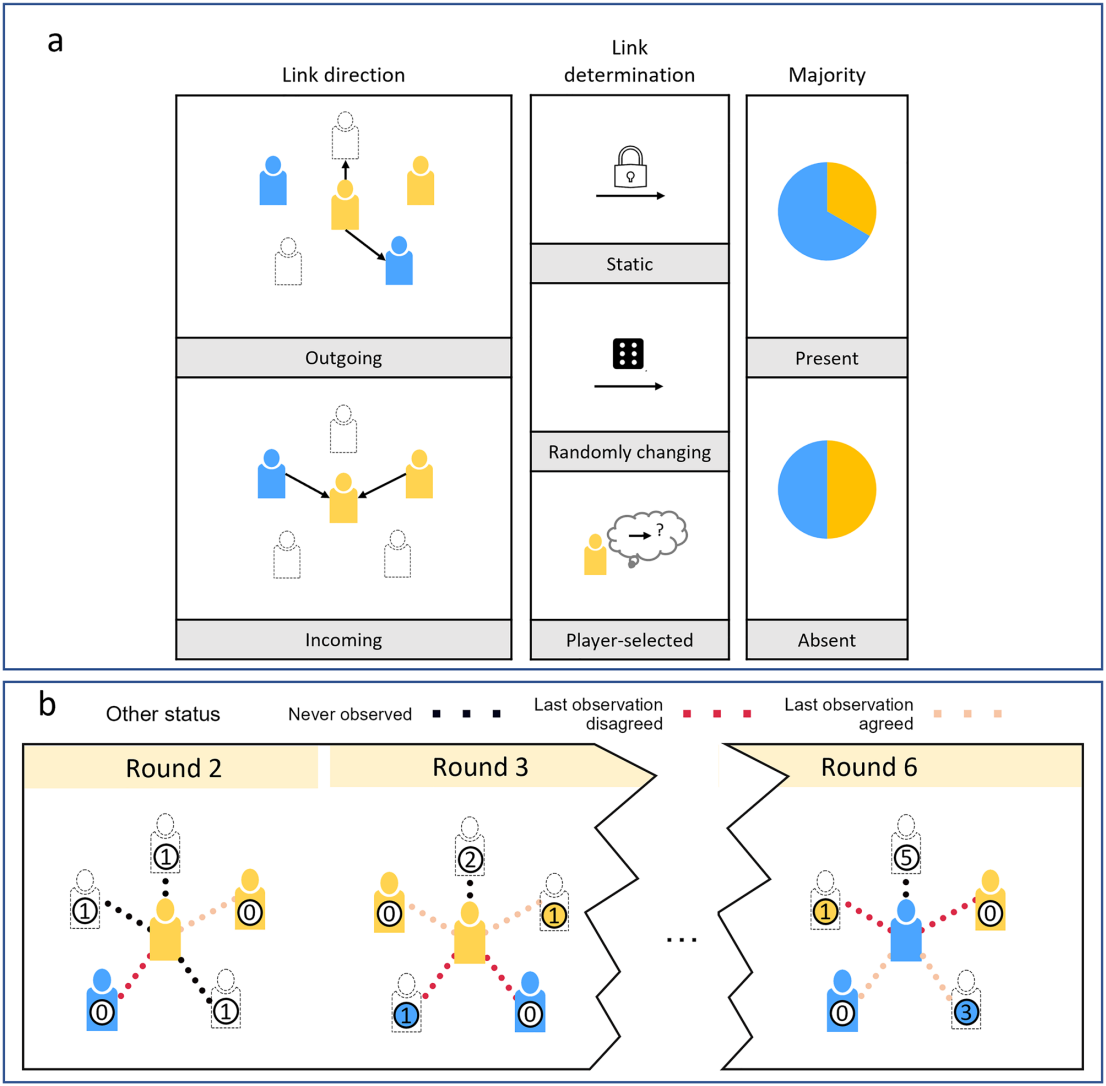
Overview of experimental design. (**a**) We varied whether links were outgoing or incoming (left); whether links were static, changed randomly in each round, or selected by the players themselves (middle); and whether a majority was present (right). (**b**) Example of a possible sequence of rounds, illustrating how the status of others and the number of rounds unseen change. Each possible link (dotted lines) can be characterized by other’s status (agreeing or disagreeing; colored lines) and how long they were unseen (inset numbers). Other players that are not currently observed are indicated with dotted icons and circles in their last seen color choice. We use the upper left player to further elaborate on the parameters used in estimations and visualized in (**b**): In round 2, this player has not yet been observed by the focal individual (black link), and this status persisted for 1 round (empty circle with number 1). In round 3, this upper left player is observed (empty circle updates to the number 0) and chose yellow (icon color), which was the same color as the focal player (orange link). In round 6, the player is again not observed by the focal player (empty icon) but was observed to select yellow the round before (yellow circle with number 1), which is the opposite color of the focal player (red link).

The present study (Fig. 1a) investigates what strategies individuals use when given the ability to select their incoming or outgoing links, whether individuals’ link-selection strategies result in the formation of clusters, and whether they benefit or hinder consensus formation. We used a color coordination task^5,14,30^, similar to previous studies^4,6^ to investigate how groups of 6 individuals with conflicting incentives for two colors (blue and yellow) seek consensus. In each round, all 6 individuals in a group selected a color, with the goal of selecting the same color as all other group members. Each individual had links with 2 other individuals at any moment in time. We varied how links were determined and compared networks with player-selected links to networks with static and randomly changing links. We also addressed whether individuals’ link selection strategies are affected by the direction of links they select by varying the direction of links in all types of networks. Moreover, we explored how individuals’ choice strategies contribute to consensus formation, as well as whether having a majority incentivized for one color speeds up consensus formation compared to groups where a majority is absent.

There are two competing possible expectations, that depend on how individuals select their communication links. We expect that networks with stable links are slower to find consensus compared dynamic random links, as polarization of votes should emerge more likely in stable communication structures. The relative speed performance of the player-selected dynamic condition should depend on the extent player-selection yields homophilous, assortative linking. The more this is the case, the slower groups with player-selected links should find consensus compared to random and static links. Finally, we also expect that an agreement will be reached faster when a majority of individuals is incentivized for one color, since individuals incentivized for a minority color will more likely observe local majorities. We preregistered our predictions on OSF (https://osf.io/j2gcn).

## Results

### Link selection

#### Individual link selection strategies

**Figure 2:**
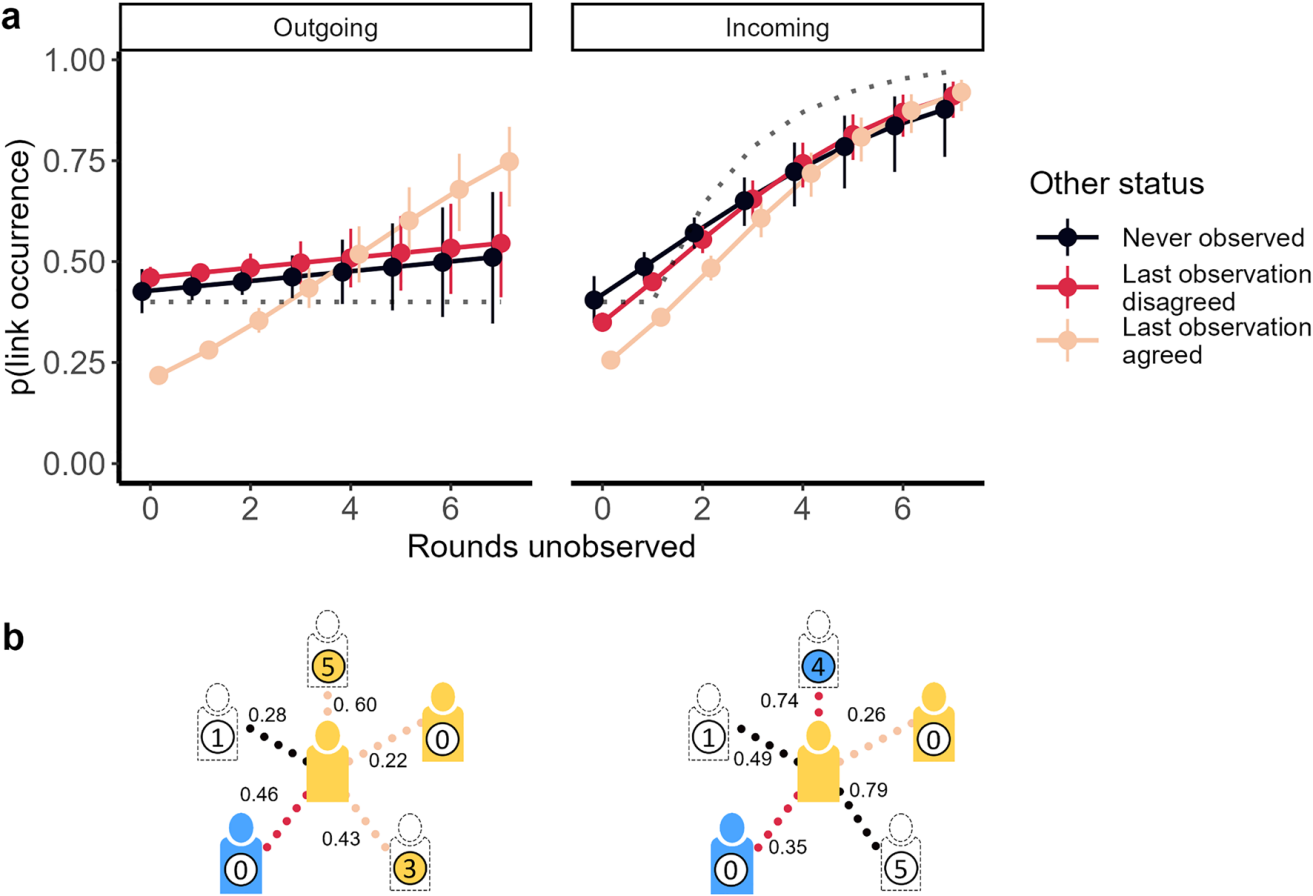
Probability of a link between a player-other pair in the player-selected link condition. Colors indicate other’s last seen choice and panels correspond to link direction. (**a**) Zero rounds unobserved indicates that an other is observed during link selection. The dotted reference line indicates the probability of observing a link by chance (since 2 out of 5 targets are selected, this is a function of p = 0.4, which is not constant for incoming links because selections and observations are mutually dependent and longer streaks of non-observation are less likely, leading to an increased likelihood of observations after long non-observation). Since 97.5% of all links were observed before an individual was unseen for 7 or more rounds, we truncated the plot at this value. Error bars correspond to 95% confidence intervals. (**b**) Example networks below each link direction illustrate situations with links colored by other status. Insets corresponding to the number of rounds unseen and the last observed color. Estimated probabilities by direction appear next to each possible link.

Our main interest was whether individuals’ link selection strategies create or avoid consensus-impeding assortment. To this end, we tested how information about others’ choices (observation status) affected link selection using a logistic mixed model predicting the probability of observing a link between a player-other pair (link probability). Overall, we find that individuals avoid like-minded others (Fig. 2).

A main effect of others’ observation status, *Wald-χ*^2^(2) = 278.116, *p* < 0.001, indicates that individuals were sensitive to whether they observed another player, and whether this other player’s last observed choice agreed or disagreed with their own previous choice. Increased link probability with increasing number of rounds unobserved indicates that individuals preferred to select others about whom they currently had no information, Wald-*χ*^2^(1) = 106.135, *p* < 0.001. The effects of observation status varied with number of rounds unseen and link direction, as indicated by 2-way interactions of status with number of rounds unseen, Wald-*χ*^2^(2) = 46.615, *p* < 0.001, status with link direction, *Wald-χ*^2^(2) = 47.479, *p* < 0.001, and number of rounds unseen with link direction, *Wald-χ*^2^(1) = 26.215, *p* < 0.001, and their 3-way interaction, *Wald-χ*^2^(2) = 12.123, *p* = 0.002. Therefore, we discuss simple effects for both link directions separately.

When individuals selected outgoing links, link-selection probabilities differed by *observation status*, *Wald-χ*^*2*^(2) = 124*.*14, *p* < 0*.*001, *number of rounds unseen*, *Wald-χ*^2^(1) = 19*.*53, *p* < 0*.*001, and their interaction, *Wald-χ*^2^(2) = 38*.*82, *p* < 0*.*001. Individuals avoided sending their choice information to others who agreed with their last color choice, compared with both those they had not observed yet, *b* =  *− *0*.*58, *OR* = 0*.*56, *Z* =  *− *7*.*35, *p* < 0*.*001, and others who disagreed, *b* =  *− *0*.*72, *OR* = 0*.*48, *Z* =  *− *10*.*69, *p* < 0*.*001, with no evidence for a difference between unobserved and disagreeing others, *b* =  *− *0*.*14, *OR* = 0*.*87, *Z* =  *− *1*.*75, *p* = 0*.*223. The link-selection probability increased with *number of rounds unobserved* for agreeing others, *b* = 0*.*34, *OR* = 1*.*40, *Z* = 7*.*79, *p* < 0*.*001, with no evidence for an effect among disagreeing others, *b* = 0*.*05, *OR* = 1*.*05, *Z* = 1*.*13, *p* = 0*.*258, and not yet observed others, *b* = 0*.*05, *OR* = 1*.*05, *Z* = 0*.*78, *p* = 0*.*436. After not observing an individual for 3 or more rounds, others’ status no longer reliably affected the probability that the player selected them, *Wald-χ*^2^(2) = 4*.*22, *p* = 0*.*121. These results suggest individuals avoid sending to agreeing others, relying on memory for others’ choices in the last few rounds.

When individuals selected incoming links, link-selection probabilities also differed by *observation status*, *Wald-χ*^2^(2) = 57*.*12, *p* < 0*.*001, *number of rounds unseen*, *Wald-χ*^2^(1) = 94*.*65, *p* < 0*.*001, and their interaction, *Wald-χ*^2^(2) = 9*.*43, *p* = 0*.*009. Like those who selected outgoing links, they also avoided observing others agreeing with their last color choice, as compared with others whom they had not observed yet, *b* = *− *0*.*46, *OR* = 0*.*63, *Z* = *− *5*.*89, *p* < 0*.*001, and disagreeing others, *b* = *− *0*.*34, *OR* = 0*.*71, *Z* = *− *6*.*28, *p* < 0*.*001, with no reliable difference between unobserved and disagreeing others, *b* = 0*.*12, *OR* = 1*.*13, *Z* = 1*.*49, *p* = 0*.*353. The link-selection probability increased with *number of rounds unobserved* for agreeing, *b* = 0*.*50, *OR* = 1*.*65, *Z* = 11*.*84, *p* < 0*.*001, disagreeing, *b* = 0*.*42, *OR* = 1*.*52, *Z* = 9*.*69, *p* < 0*.*001, and unobserved others, *b* = 0*.*34, *OR* = 1*.*40, *Z* = 4*.*57, *p* < 0*.*001. Still, after not observing others for 4 or more rounds, others’ status no longer reliably affected the probability that the player selected them, *Wald-χ*^2^(2) = 1*.*17, *p* = 0*.*558. Thus, similar to what was found for outgoing links, individuals also avoid observing agreeing others, with an influence of others’ choices from the last few rounds.

Interestingly, when we directly compare *link direction* conditions, individuals in the outgoing links condition more likely selected currently disagreeing others (number of rounds unobserved equal to zero), *b* = 0*.*46, *OR* = 1*.*59, *Z* = 6*.*17, *p* < 0*.*001, but less likely selected currently agreeing others, *b* = *− *0*.*21, *OR* = 0*.*81, *Z* = *− *2*.*54, *p* = 0*.*011, and equally likely observed others who had not been observed yet, *b* = 0*.*09, *OR* = 1*.*09, *Z* = 0*.*52, *p* = 0*.*606, compared to the incoming links condition. In line with our expectations, individuals avoided disagreeing others more when they selected their observations than when they selected recipients, whereas they avoided agreeing others more when they sent their choices than when they selected who they observed. In sum, individuals preferentially avoided linking with those who agreed with them, and thus avoided creating assortment of choices (robustness checks in Supplementary Sect. 3).

#### Group assortment

As expected, average network assortment differed by our experimental factors (see also Supplementary Fig. 9). Network assortment is a measure of how likely nodes of the same type (i.e., color choice) are to be connected to one another. It is based on the proportion of same-choice links in each round (see Supplementary Methods). Choice-based network assortment, averaged across rounds for each network, differed between *link determination* conditions, *F*(2,232) = 19*.*64, *η*^2^_*G*_ = 0*.*145, *p* < 0*.*001. Assortment was overall lowest when links were player-selected, both compared to static networks, *b* = *− *0*.*10, *d* = *− *1*.*00, *t*(232) = *− *6*.*27, *p* < 0*.*001, and randomly changing links, *b* = *− *0*.*06, *d* = *− *0*.*54, *t*(232) = *− *3*.*33, *p* = 0*.*001. Also there was less assortment among randomly changing than static links, *b* = *− *0*.*05, *d* = *− *0*.*46, *t*(232) =  *− *2*.*94, *p* = 0*.*004. An effect of *link direction*, *F*(1,232) = 6*.*96, *η*^2^_*G*_ = 0*.*029, *p* = 0*.*009, indicates less assortment for outgoing links. There were no differences depending on the presence of a *majority*, *F*(1,232) = 0*.*92, *η*^2^_*G*_ = 0*.*004, *p* = 0*.*338, or any 2-way interactions of the factors (all *η*^2^_*G*_ < 0*.*004 and all *p* > 0*.*33).However, there was evidence for a 3-way interaction of link determination, link direction, and majority, *F*(2,232) = 3*.*35, *η*^2^_*G*_ = 0*.*028, *p* = 0*.*037: Player-selected links resulted in lower assortment than static links across conditions (all *d* > 0*.*74, all *p* < 0*.*016), whereas comparisons to random link determination were more mixed (see Supplementary Sect. 3.2). Thus, individuals’ link selection strategies enabled them to create lower assortment levels than in static networks, but not consistently lower assortment than in random networks.

Assortment between outgoing and incoming links did not reliably differ when links were player-selected, irrespective of whether there was a majority present, *b* = *− *0*.*01, *d* = *− *0*.*14, *t*(232) = *− *0*.*45, *p* = 0*.*655, or absent, *b* = *− *0*.*02, *d* = *− *0*.*24, *t*(232) = *− *0*.*68, *p* = 0*.*497. Thus, link selection differences between link directions (i.e., selectively avoiding agreeing others for outgoing and disagreeing others for incoming) did not result in different network-level properties. In contrast to the differences in assortment seen when considering individuals’ choices, we do not find any of the above differences when we consider average assortment according to the incentivized color (Supplementary Sect. 3.2, Supplementary Fig. 10). This likely reflects that individuals did not have this information and choice dynamics rapidly distorted the incentivized color, so that choices only reflect the incentivized color noisily.

### Choice behavior and agreement

#### Individual choice behavior

**Figure 3:**
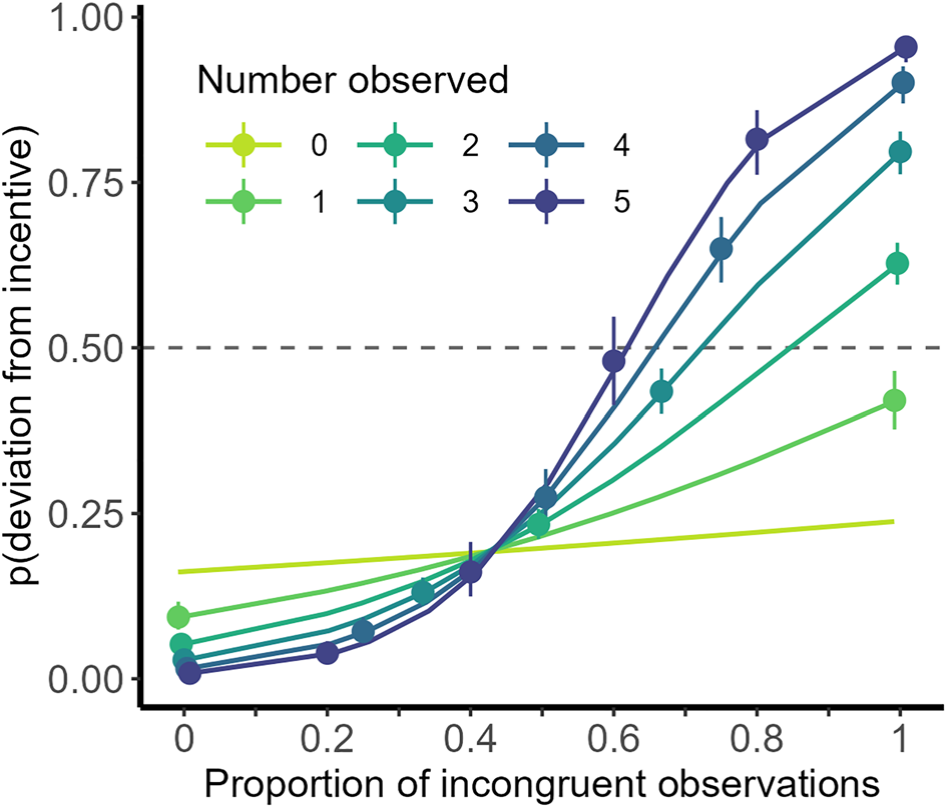
Probability of deviating from incentive by number and proportion of deviating observations. Both the proportion of incentive-incongruent observations and the number of total observations affect the probability that individuals deviate from their incentivized option. Points indicate estimates for possible proportions. Error bars correspond to 95% confidence intervals.

Earlier research suggests that individual choices should be sensitive to the observed majority^4^. In line with this expectation, a logistic mixed model shows that individuals were more likely to deviate from their incentivized option the higher the proportion of incentive-incongruent choices they observed in a given round (a unit increase corresponding to 10% more incentive-incongruent votes observed), *Wald-χ*^2^(1) = 625*.*479, *p* < 0*.*001. The likelihood of deviating also increased with the number of observations in this round, *Wald-χ*^2^(1) = 6*.*606, *p* = 0*.*010, and the interaction between these two factors, *Wald-χ*^2^(1) = 151*.*912, *p* < 0*.*001 (Fig. 3). This indicates that individuals’ decisions were not only impacted by the proportion of incentive-incongruent choices, but also by the absolute number of incentive-incongruent choices observed. Surprisingly, there was evidence for a small effect of the proportion of incongruent votes in the absence of observations, *b* = 0*.*05, *OR* = 1*.*05, *Z* = 1*.*99, *p* = 0*.*047. This could indicate that also past observed choices had a small influence on deviation behavior. Additionally replicating Gaisbauer et al*.*^4^, participants exhibited inertia inasmuch that a deviation from their preference in the previous round was predictive of deviating again, *b* = 1*.*345, *OR* = 3*.*838, Wald *Z* = 21*.*826, *p* < 0*.*001 (Supplementary Fig. 11a).

These results were robust to the inclusion of experimental factors and aggregate indicators for deviations and observations on the individual and group level (Supplementary Sect. 4). This extended model shows that yielding to a local majority was less pronounced in dynamic networks, *Wald-χ*^2^(2) = 22*.*065, *p* < 0*.*001, for outgoing links, *Wald-χ*^2^(1) = 6*.*599, *p* = 0*.*010, and, marginally, when a majority was present in the network, *Wald-χ*^2^(1) = 3*.*552, *p* = 0*.*059. Overall, individuals responded to their local observations by compromising their incentivized preference when confronted with a disagreeing majority.

#### Consensus performance

Out of all 244 networks 68.85% reached a consensus, 25.82% dropped out, and 5.33% did not converge within 50 rounds. Thus, individuals were generally successful at reaching consensus, but there was also a substantial amount of drop-out, in part due to the technical challenges of this group paradigm.

##### Convergence probability and speed

**Figure 4:**
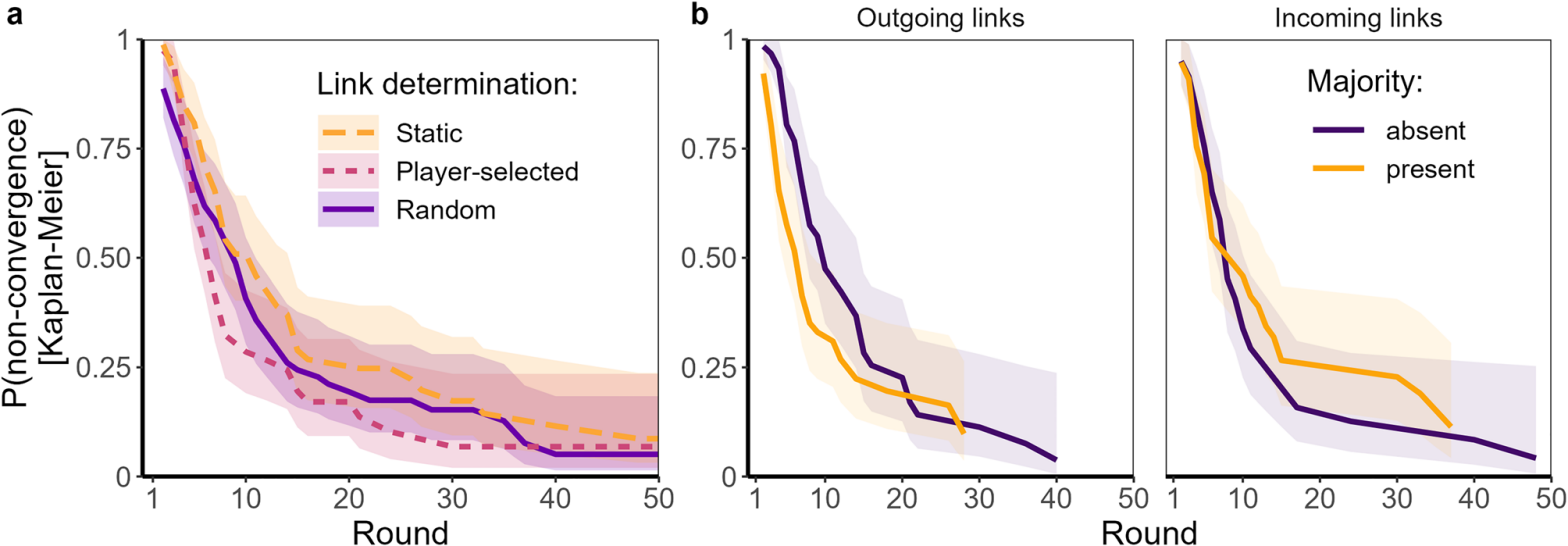
Differences in convergence speed. Survival probability as a function of (**a**) link determination and (**b**) link direction and the presence of a majority. Lines correspond to the probability of non-convergence in a given round. Shaded regions correspond to 95% confidence intervals.

Dependent on whether individuals selected links to agreeing others or not, we expected that player-selected links impede or benefit consensus speed relative to static and randomly changing networks. Link-determination conditions differed in convergence speed (Fig. 4a), *LR*-*χ*^2^(2) = 7*.*328, *p* = 0*.*026. Networks where participants selected their links converged faster compared to static communication networks, *b* = 0*.*53, *HR* = 1*.*70, *p* = 0*.*020, but we do not find evidence for faster convergence compared to when links changed randomly, *b* = 0*.*27, *HR* = 1*.*31, *p* = 0*.*408. There was also no evidence for a convergence speed difference between randomly changing and static links, *b* = 0*.*26, *HR* = 1*.*30, *p* = 0*.*444. However, the fact that our tests did not reliably detect the HRs around 1.3 could reflect limited statistical power. Notably, the probability of early dropout was increased when individuals selected their links, possibly because the users had to perform more actions in this condition, which could have decreased our overall statistical power making results about the player-selected condition less reliable (see Supplementary Sects. 2 and 4 for Figures and robustness checks). Overall, network dynamics improved convergence speed and the benefit for dynamic links likely reflects link formation strategies.

Contrary to our expectations, the presence of a majority did not generally affect convergence speed (Fig. 4b), *LR*-*χ*^2^(1) = 0*.*772, *p* = 0*.*380, but did so as a function of link direction, *LR*-*χ*^2^(1) = 5*.*807, *p* = 0*.*016: Networks converged faster in the presence of a majority when links were outgoing, *b* = 0*.*52, *HR* = 1*.*68, *p* = 0*.*018, but not incoming, *b* = *− *0*.*24, *HR* = 0*.*79, *p* = 0*.*287.

##### Majority consensus

As expected, among converged networks the majority won in 83.9% of cases; *CI*_95%_ = [74*.*5; 90*.*9], *p*_binomial(87*,*0*.*5)_ < 0*.*001(Supplementary Figs. 12 and 13).

Our preregistered logistic mixed model predicts individuals’ probability of choosing blue in the final round as a function of (a) whether there was any majority and its respective color (majority color), (b) link determination, (c) link direction, and (d) each individual’s incentive, including random intercepts and random slopes for individuals nested in networks. Consistent with our prediction, the majority-color affects individuals’ probability of choosing blue in the final round, *Wald-χ*^2^(2) = 36*.*094, *p* < 0.001. We also find some support for our prediction that this majority-color effect varied by link determination, *Wald-χ*^2^(4) = 11*.*889, *p* = 0*.*018. Individuals selected the majority-color more likely than the other color if links changed randomly, *χ*^2^(2) = 22*.*78, *p* < 0*.*001, or were self-selected, *χ*^2^(2) = 36*.*19, *p* < 0*.*001, but evidence is only marginal in static networks, *χ*^2^(2) = 5*.*88, *p* = 0*.*053 (likely reflecting larger uncertainty, Supplementary Sect. 4). Overall, the majority could benefit more reliably from dynamic links (Supplementary Fig. 14), but this finding is not fully robust to other estimation methods (see Supplementary Sect. 4). This benefit may be explained further by specific characteristics of static networks (for exemplary networks see Supplementary Sect. 5).

## Discussion

Individuals in a color coordination task strategically select their communication links, mainly observing and sending to others they have not recently observed or who disagree with them. Hence, individuals avoid forming polarized clusters that could have emerged from assorting with like-minded others. Like individuals without additional incentives, they respond to their local majority^4,31,32^, but also to the absolute number of incongruent choices they observe, reflecting the idea of complex contagion^2^. Overall, both link selection and choice strategies allow individuals to reach consensus relatively quickly compared to static networks.

Individuals shape their local information to benefit consensus formation by avoiding clusters, structural imbalance, and potentially also by tackling uncertainty and considering the instability of choices. Not only do individuals appear to avoid clusters, their strategies also avoid misrepresentation of majorities. In line with our expectations, the majority wins most of the time, but is less reliably at an advantage when links are static rather than dynamic. Whereas in static networks, the fixed structure and zealot individuals can create situations in which the majority is overthrown^11,14,16^, dynamically changing links reduce such undemocratic outcomes.

Link selection may even facilitate consensus formation beyond what would be expected from random dynamics. Link selection could provide all individuals with an accurate proxy of the global distribution of color choices, reducing social uncertainty^33^. Individuals selectively observe those who they currently have little or no information about, and selectively inform others who they disagree with or have not yet observed. These findings are also reflected in individuals’ self-reported strategies (Supplementary Sect. 6). The tendency to avoid selecting those who were previously observed could reflect that repeated observations of the same individual offer little additional information. Furthermore, the observed link selection strategies also account for the fact that others’ choices are dynamic. Relying on memory of others’ choices for the most recent rounds of play avoids reliance on outdated color choices, exemplifying the adaptivity of limited memory and forgetting in changing environments^34^.

Contrary to our expectations, incentivizing a majority for one outcome reliably increased consensus speed only when links were outgoing. This could be because individuals were less likely to form clusters when links were outgoing. In addition, outgoing links could expose the minority to a larger number of deviating choices, since in this condition the number of observations in any given round could vary between 0 and 5, whereas for incoming links the number of observations per round was fixed to 2 by design. These findings highlight the role of differences in degree centrality for the speed^14^ and outcome^6^ of consensus formation, also reported in the context of problem solving^35^.

The supposed formation of clusters or echo-chambers based on individuals’ opinions^8,21,36–38^ does not generalize to our consensus formation task, echoing previous work questioning the relevance of echo chambers^12,13^. Since link formation strategies do not lead to opinion-based cluster formation, this raises the question of under which conditions echo-chamber like clusters *do* form. First, there can be pre-existing clusters based on spatial association and other individual features, such as demographics^20^, externally limiting information search. Moreover, in the present study all individuals were strongly incentivized to agree. Larger consensus costs for forgoing ones’ preference^1^ or a weaker incentive for agreement could alter individuals’ selection strategies. When consensus is not required and splitting the group is an acceptable outcome, selecting like-minded others could increase the chances to get the group to agree on their preference at the possible cost of forming a sub-group. Finally, the current study design did not reflect that interactions with others who disagree can be more costly or effortful than interactions with likeminded others, for instance, because they require dissonance reduction^39^. Thus, individuals might break links with those who strongly disagree with them^21,29^ to avoid these costs, similar to past work showing that cooperative individuals form clusters to avoid the costs of interacting with defectors^23–25^. Beyond individual strategies in such other settings^40^, recommendation algorithms may create echo chambers by linking structurally similar individuals^41^. It is unclear whether such external influences can be counteracted by individual strategies. Although other conditions may exist where clusters form, situations where consensus is highly valued could constitute a crucial boundary condition to the dangers associated with homophily and polarization.

Importantly, the conclusions drawn from the present study may only generalize to certain decision situations and networks. Firstly, many real-world networks—especially in the online realm—are very large and exceed our group size by orders of magnitude, and individuals could apply other strategies that do not avoid clustering in these contexts. Constraints of memory or attention, as well as pre-existing network structures could prevent individuals from accurately assessing others’ choices, or require them to rely on external cues about other opinions. Still, this study illustrates that polarized communication network structures are not due to individual strategic deficits. Secondly, increased dropout among networks with player-selected links could reflect technical demands and the additional actions individuals needed to perform. Even though individuals’ choice and link formation strategies are likely unaffected by this, it may affect our conclusions about the benefits of network control. Intriguingly, the dropout may hint at the cost of control over ones’ network: selecting communication partners could sometimes entail opportunity costs that outweigh the benefits. Relatedly, in real world networks connections to others may be costly since one needs to invest effort into connecting with others^42^ or pay for information^43^. Although online-mediated communication may have reduced the cost of obtaining and sending information^44^, the motivation to invest time and effort may vary among individuals. To address these limitations, future research should more closely investigate how communication costs may impact consensus speed and outcomes.

By improving our understanding of individual-level link selection strategies we contribute to a timely understanding of collective behavior^44^ and the threat of political polarization^10^. Fortunately, people motivated for consensus spontaneously avoid connections to those who agree and, thus, dodge the threat of polarization and echo chambers. In the end, similar to the results of Sherif’s notorious robbers-cave study^45^, a common goal can foster strategies that limit polarization and conflict in social networks. Thus, making incentives for consensus more salient could lead individuals to get in contact with others who disagree^9^—similar to politically interested individuals who seek more diverse information^46^—providing a possible mechanism to avoid echo chambers and network polarization.

## Materials and methods

### Design

We used a networked color coordination paradigm to study how control over ones’ communication network affects consensus formation in the presence of different preferences. We manipulated individuals’ preferences by incentivizing each individual to prefer a specific color (i.e., to be opinionated^4,6,14^). Players received an additional bonus of 0.50 USD if the network converged on their color. Participants were unaware of other players’ incentives. Network links determined which other players’ color choices a player could observe. We aimed to understand the strategies that individuals use to determine their interaction partners and to disentangle the effects of dynamic network change and individuals’ control over the network structure. To this end, we compared networks differing in their link determination such that links to others were either *player-selected*, *randomly changing* (link dynamics without players’ control) or *static* (neither control nor dynamics). We also varied the *link direction* (outgoing vs. incoming) to assess whether link selection strategies and collective outcomes are sensitive to whether individuals control who they can observe (incoming links) who is able to observe them (outgoing links). Depending on link direction, players’ in-degree or outdegree respectively was always kept constant at 2. Finally, to assess whether and when individual strategies are able to topple or support a numeric majority, we varied whether a *majority* was *present (4 individuals incentivized for one color, 2 for the other)* or *absent* (3 individuals incentivized for each color). These aims resulted in a 3 (link determination: static vs. randomly changing vs. player selected) × 2 (link direction: outgoing vs. incoming) × 2 (majority: present vs. absent) design.

### Participants

We recruited 2309 participants in 385 networks on the online crowd-sourcing platform Amazon Mechanical Turk (MTurk, www.mturk.com). We ensured technically that individuals did not participate multiple times or in earlier related studies^4,6^. Overall drop-out rates of 9.6% after being grouped and 12.7% failing to reach the final page were in line with comparable online studies^4,6,47^. In round 1, we lost 29.1% (112) of all networks due to participants missing the start. In the first round, 6 networks converged and 22 dropped out, although all individuals made a choice, probably due to technical problems. As pre-registered, these 28 networks were included for determining the sample size. Deviating from our pre-registration (including all networks with at least 6 choices), these networks were not considered further in our analyses but their inclusion does not change the pattern of results. This resulted in a total sample of *N* = 13,284 observed rounds of *n*_*ind*_ = 1464 unique individuals (50.41% self-identified as female, *M*_*age*_ = 37*.*34 years and *SD* = 11*.*15 years) in *n*_*network*_ = 244 (of 270 planned) networks. 245 networks would allow us to detect small to medium network-level mean-differences (*η*^2^_*G*_ ≥ 0.038; instead of *η*^2^_*G*_ ≥ 0.022), including 3-way interactions (df = 2) of our experimental factors, and power on all levels mainly depends on this highest level^48^ (see Supplementary Sect. 2).

Participants received a reward of 2.00 USD for participation and earned an average performance bonus of USD 0.84 (*SD* = 0*.*61). They needed an average of 15*.*34 min (*SD* = 5*.*52 min) to complete the study. The study adhered to the Declaration of Helsinki, relevant laws, and institutional guidelines, as certified by IRB of the University of Konstanz. The University of Konstanz IRB approved the study. All participants gave informed consent.

### Procedures

The experiment was conducted online via the software oTree^49^ and the procedures are comparable to previous studies^4,6^. After informed consent, participants were presented a CAPTCHA to screen out automated scripts, followed by detailed instructions and a comprehension test. For the main task, participants were placed in groups of 6 players. Participants entered a waiting page where they received a bonus payment of approximately 0.05 USD per minute they could not be assigned to a group, up to a total of 10 min after which they could leave the study. Once grouped, participants were instructed to find a group-wide consensus by unanimously agreeing on one of two colors (blue vs. yellow) as fast as possible within 50 rounds. In each round, players chose either blue or yellow. If the network converged on one color, each player received a bonus payment which started at 1.00 USD and decreased by 0.02 USD increments per round. In conditions where they had control over their network links, participants additionally selected links to 2 other players in their group whose color choice they would observe (when links were incoming) or who would observe their color choice (when links were outgoing) in the next round.

To create conflicts, each participant received an additional reward of 0.50 USD if the group came to consensus on one particular color. Colors were randomly assigned to participants with the number of participants assigned to each color determined by the majority condition (3:3 or 4:2). If a participant dropped out of the study, we stopped the experiment for that group. The abandoned group members, and also all others completing the task, received a base payment of 2.00 USD and any payments for their waiting time, regardless of success.

Participants received feedback on their total earnings after the main task, completed a short survey on satisfaction with the task and potential strategies they had implemented, responded to a social value orientation scale, and were debriefed. For a view of the participant screen in the task, see Supplementary Fig. 4.

### Data analyses

All analyses were conducted in R-4.1.2^50^ (model descriptions and tables see Supplement). To interpret interaction effects, we report estimated marginal means (Wald Z or *t-*test*s)*, as well as Wald *χ*^2^ tests for simple main effects and interactions. Models account for drop-out by using the data of all individuals and networks (including those that dropped out after round 1), retaining statistical power on the highest level of analysis (Supplementary Sect. 2; Bayesian models additionally provide uncertainty about estimates given the data, see Supplement).

#### Link selection

To explore individuals’ link selection strategies in the player-selected condition, we modelled the probability of each possible link between all five possible player-other pairs with a logistic mixed model. We analyzed a total of *n*_*ind*_ = 474 individuals in *N* = 3408 individual rounds (17,004 possible link observations between each of the 5 possible pairs). We excluded the first choice of all participants, since in the initial round they did not have any information about others. Link probability was modelled as a function of properties of the possible links in each pair (Fig. 1b), including (1) 3 levels of other’s observation status (*unobserved* or other’s last observed choice *agreeing* or *disagreeing* with the player’s last choice), (2) the number of rounds that the other individual had been unobserved, (3) link direction on the network level, and (4) their cross-level interactions. We included random effects for each other and each possible player-other pair across rounds, and random slopes for the effect of the number of rounds the other was unseen (see Supplementary Table 2 and Supplementary Sect. 4, including higher-level contextual effects).

#### Assortment

We compared average network-level assortment with classical ANOVAs.

#### Choice behavior

Using logistic mixed models, we modelled the probability that individuals deviated from their incentivized color as a function of (1) round number, (2) whether their previous choice deviated from their incentivized color, (3) the proportion of incentive-incongruent choices among visible others, (4) the total number of others observed, and (5) the interaction of proportion and total number (all at individual round level). We included random intercepts for individuals, as well as random slopes for round and the proportion of deviations observed (Supplementary Table 4 and Supplementary Sect. 4, including higher-level contextual effects). We excluded the first choice of participants, since initially they did not have any information about others.

#### Consensus probability and speed

To analyze the time dynamics of consensus formation on the network-level, we used Cox Proportional Hazard survival regressions (Supplementary Sect. 4). As pre-registered, we predicted the hazard for reaching group-level consensus as a function of our experimental factors: *link determination* (*static* vs. *changing randomly* vs. *player-selected*), *link direction* (incoming vs. outgoing), and majority (*absent:* 3:3 incentives for each color vs. *present:* 4:2 for one color), as well as all interactions between them. To account for drop-out, we included networks that dropped out of the study as right-censored data.

#### Majority consensus

To analyze the impact of a majority on the final choice we report a pre-registered logistic mixed model (Supplementary Table 9). We predicted the probability of individuals’ blue choices in the final round as a function of our experimental factors: (1) link determination (static vs. randomly changing vs. player-selected; effect coded with static as reference), (2) link direction (incoming vs. outgoing, with incoming as reference), (3) a 3-level factor for the presence and direction of a majority (*absent* vs. 4 blue, 2 yellow vs. 4 yellow, 2 blue), (4) all interactions between them (all at network level) and (5) individuals’ incentivized color (blue vs. yellow, individual level). We included random intercepts for the network and random effects for the incentivized color.

## Supplementary Information

Supplementary Information.

## Data Availability

All data, code, and materials have been made publicly available at OSF and can be accessed at osf.io/q25ah.
