# Supplementary material for: Humans strategically avoid connecting to others who agree and avert the emergence of network polarization in a coordination task
Source: Sci Rep. 2023 Jul 12;13:11299. doi: 10.1038/s41598-023-38353-w (PMC10338681; doi:10.1038/s41598-023-38353-w)
Supplement: Supplementary file 1 — Supplementary Information. [file 41598_2023_38353_MOESM1_ESM.pdf]

Supplementary materials:
Humans strategically avoid connecting to others who agree and avert the emergence of network polarization in a coordination task

Nico Gradwohl[1,3,4,*], **Ariana Strandburg-Peshkin**[2,3,4], **and Helge Giese**[1,3,5]

[1]Department of Psychology, University of Konstanz, Germany
[2]Biology Department, University of Konstanz, Germany
[3]Centre for the Advanced Study of Collective Behaviour, University of Konstanz
[4]Department for the Ecology of Animal Societies, Max Planck Institute of Animal Behaviour, Konstanz, Germany
[5]Charité – Universitätsmedizin Berlin, Germany
[*]ngradwohl@ab.mpg.de

Contents

1 Agent-based simulations **2**

2 Supplementary methods **4**

2.1 Task interface 4

2.2 Drop-out and exclusions 5
Effects of drop-out on power • Dropout speed and probability

2.3 Statistical models 7
Link selection • Choice behavior • Majority consensus • Survival models of convergence and drop out

2.4 Measures 9
Assortment • Social value orientation

3 Link selection **10**

3.1 Individual link selection strategies 10
Link selection limited to immediate observations

3.2 Group Assortment 14

4 Choice behavior and agreement **15**

4.1 Individual choice behavior 15

4.2 Consensus performance 19
Convergence probability and speed • Majority consensus

5 Specific networks **25**

6 Self-reported link-selection strategies **27**

References **28**

1 Agent-based simulations

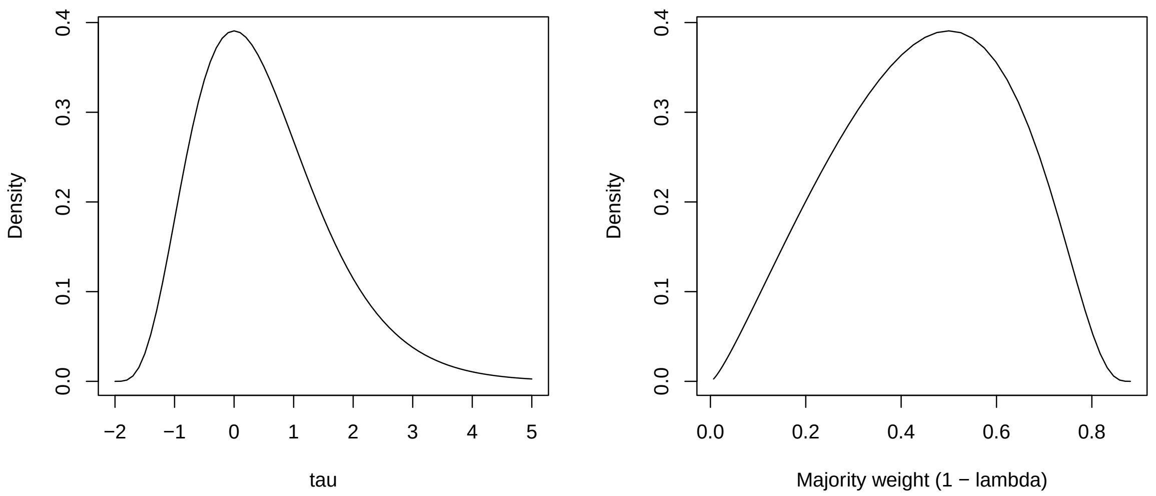

Supplementary Figure 1. Weights used in simulations. (A) Distribution of τ and (B) its logistic transformation into the majority weight $1-\lambda$.

Prior to data collection we conducted a set of agent-based simulations. Basic results can be found on OSF (`https://osf.io/q25ah/`). We had networks of 6 agents with a fixed in- or outdegree of 2 interact for a maximum of 50 rounds. Individual agents made choices between option 0 (i.e., yellow) and option 1 (i.e, blue) as a function of their preference and the local majority among those they observed.

Like in the experiment, agents' observations were determined based on 2 factors: link direction and mode of link determination. Links were either incoming or outgoing (corresponding to observing the choices of others or sending one's choice to others respectively). The links in each round were either determined randomly once in the beginning (static), randomly in each round (randomly changing), or based on agents' decisions (agent-selected). The in- or outdegree was always kept constant at 2 (i.e., 2 incoming or 2 outgoing links). Moreover, we varied whether one preference was more frequent than the other (4:2) or not (3:3), as in the experiments. When there was a majority option, it was always defined to be option 1 (blue).

Agents' choices were determined based on the probability that the agents would stick to their preferred option (0 or 1; a_i), frequency-based learning[1], and a noise parameter (η). In the absence of any majority, individuals selected their preferred option (with some noise), whereas their probability to rely on a majority signal (if present) was governed by the majority weight $1-\lambda$. The preference weight λ was derived from the parameter τ, which was drawn from a gamma distribution (see Supplementary Figure 1).

$$\lambda = \frac{1}{1+exp(\tau)}$$

The goal was to more strongly sample values of τ around zero ($a_i = 0.5$) and below but also include high values of τ, yielding some strong, mostly intermediate, and some weak majority weights.

Next, we varied agents' links selection strategies in the selective condition: we considered 3 basic strategies

- homophily: a preference to connect to others who showed a choice congruent with the agent's preference, likely breaking links to different others
- heterophily: a preference to connect to others who showed an opinion opposite to the agent's preference, likely breaking links to similar others
- uncertainty: a preference to connect to those who one does not have information on, leading to more likely breaking links to currently observed others

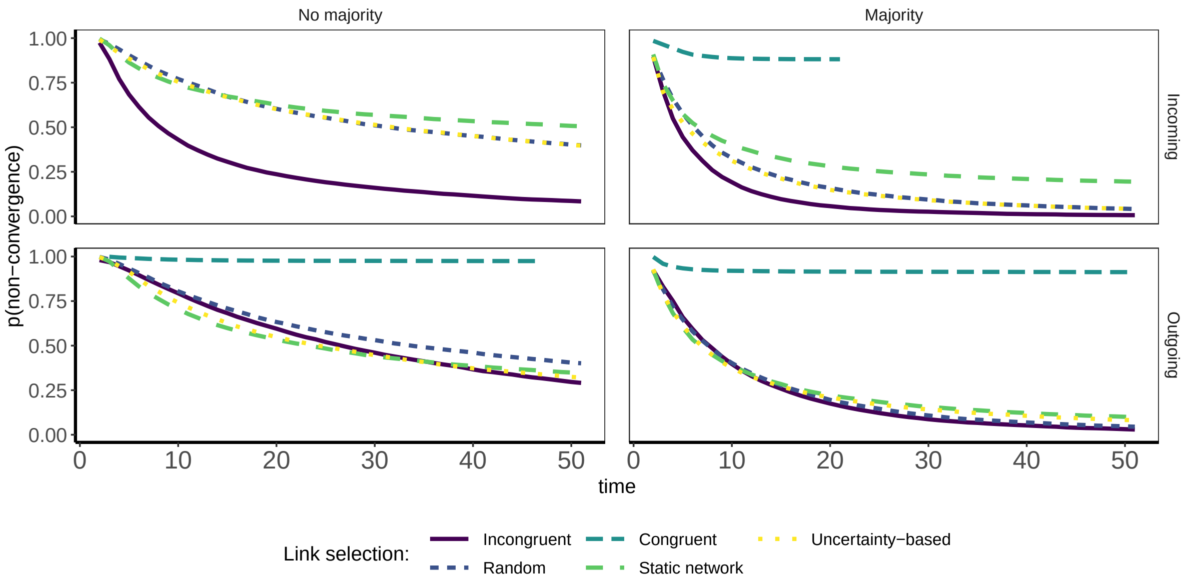

Supplementary Figure 2. Probability of non-convergence in our agent-based simulations as a function of link selection strategies by condition. Selecting congruent others prevents convergence, whereas selection of incongruent others mainly speeds up convergence for incoming links.

We examined networks in which all individuals used the same strategy to determine their links, and networks in which individuals who preferred option 1 used a different strategy than individuals who preferred option 0 over option 1 (see Supplementary Figure 3). Moreover, we varied the degree of noise in the choice determination (2 steps: 0 noise or a 0.1 chance to make a random choice).

Results are based on 10,000 networks for each parameter combination. We ran each iteration for 50 rounds, and determined as network-level outcomes whether the network converged in a given round, and if so in which. In each round, we first determined agents' observations. Based on their observations and strategy, agents selected their links. Then agents selected their color based on their incentivized preference and any observed majority (see above).

The main simulation results can be seen in Supplementary Figures 2 and 3. Blue has been arbitrarily coded as 1, with yellow as 0. Importantly, when all agents selected congruent others, networks did not converge or converged slower (Supplementary Figure 2. Especially for incoming links (fixed in-degree) selecting incongruent others sped up consensus formation. Randomly changing and uncertainty-based links mostly provided an advantage over static networks for incoming links. Thus, individuals should generally avoid selecting congruent others, as they did in the experiment.

The absent advantage for any link dynamics among outgoing links is at odds with our experimental results, suggesting that some of the assumptions in our simulations were not met by the data. The simulations presented here, for instance, did not consider memory of others' past choices. Selectively targeting those who disagreed previously, while ignoring those who agreed, could have provided an additional advantage.

Beyond consensus speed, link selection strategies could sometimes affect the consensus outcome (Figure 3), as selection of congruent others impeded consensus even in the presence of a majority. There were no marked differences for the other strategies however. Interestingly, when blue agents selected incongruent others and yellow agents selected congruent others (a) for outgoing links consensus on blue became more likely, whereas (b) for incoming links consensus on blue became less likely. This asymmetry suggests that individuals should rely on different strategies dependent on whether they send their choice to others (outgoing links) or decide who to observe (incoming links). If one faction systematically observes those who disagree, whereas the other does not, consensus will be biased towards the faction who does not observe those who disagree. This is consistent with recent mean-field models, suggesting the same asymmetry for dynamics in larger networks[2].

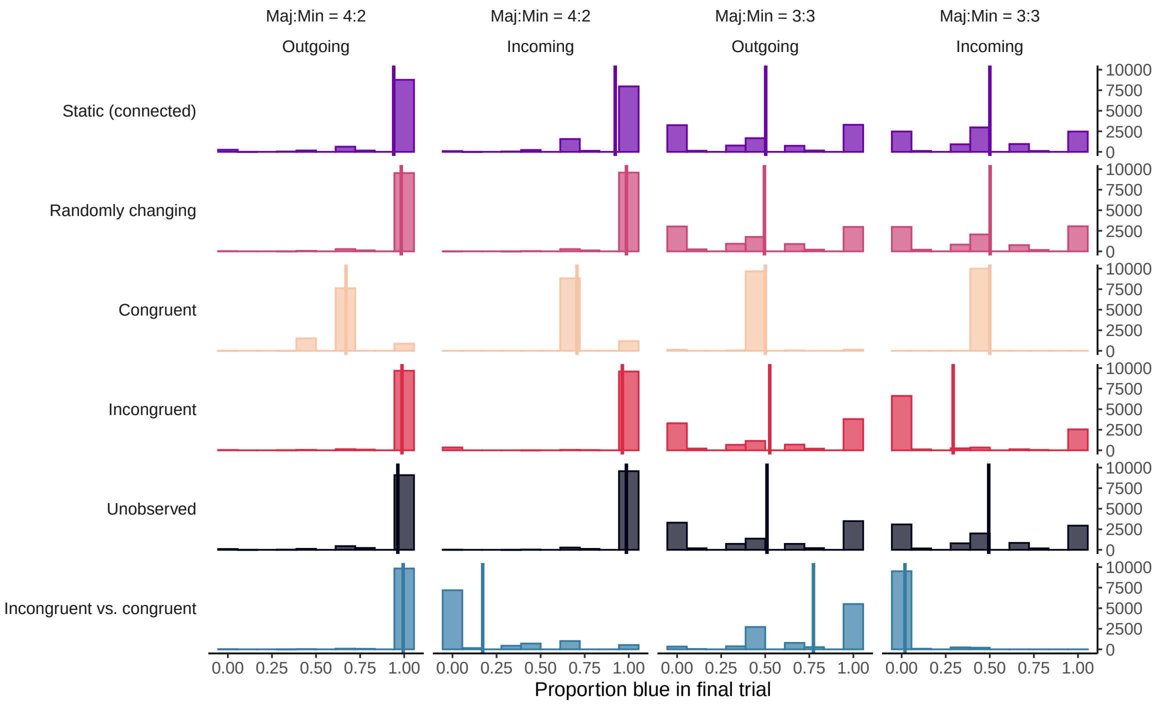

Supplementary Figure 3. Distributions of the proportion of blue (coded as 1) choices in the final round in our simulations as a function of link direction, whether there was a blue majority (columns), and different link-selection strategies (rows). This strategy impedes consensus irrespective of link direction and majority. When blue (1) individuals select incongruent others, whereas yellow (0) individuals select congruent others (Incongruent vs. congruent), consensus on blue becomes more likely for outgoing links. Conversely, it becomes less likely for incoming links, even if blue is in the majority. Vertical lines correspond to the mean proportion of blue.

2 Supplementary methods

2.1 Task interface

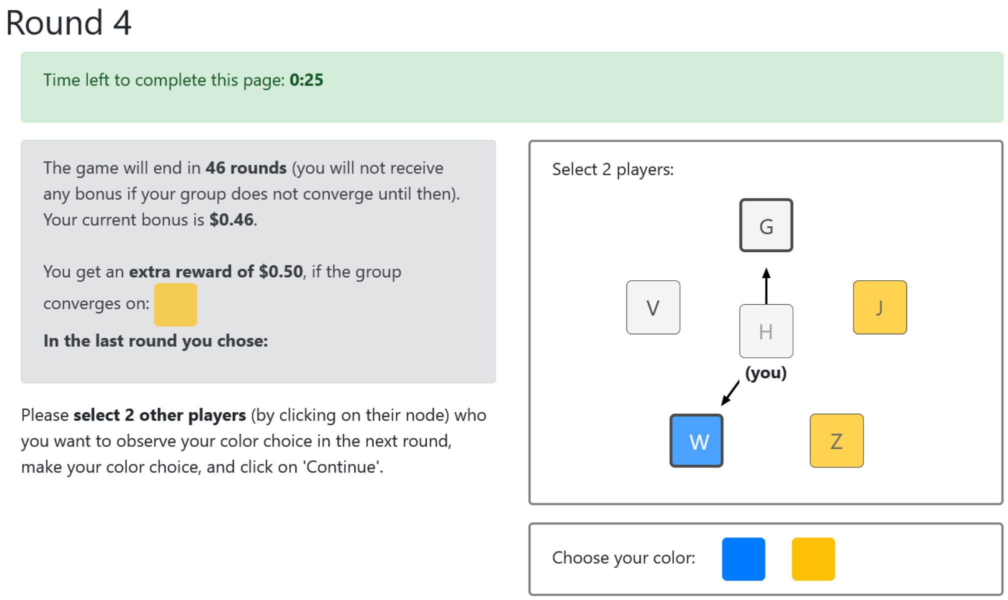

Supplementary Figure 4. Example task interface. Links were player-selected and outgoing.

2.2 Drop-out and exclusions

The online setting created drop-out during the main task. This affected the other participants in the group and warranted the exclusion of certain participants. In line with our pre-registration, we excluded participants during data collection without compensation at different stages of the study. The data from all other participants were used in subsequent analyses.

Before being assigned to a network, participants were excluded if (1) they entered a response into a so-called honeypot, which is invisible to humans but will be targeted by simple bots, (2) they failed to pass one of 2 CAPTCHAs (choosing 4 pictures with cars out of 8 pictures in total or choosing 4 dogs out of 8 pictures in total. The latter of which are displayed only after non-compliance with an instructional check.); (3) they failed to press one out of six differently colored buttons matching a target color within five seconds; (4) they submitted incorrect answers to a five-item comprehension test regarding the instruction of the main task for five times; (5) they did not have the browser window displaying the page collecting participants for network formation in focus for more than 30 seconds in total. After participants had been assigned to a group, they had to leave the study if (6) our server side could not register a participant's response for the main choice task during any round within 25 seconds (user times out).

In our analyses, we used the data of networks and participants who either completed the study or dropped out prematurely after they were assigned to a group. Thereby, we required that a group has played at least 1 round, that is, all participants in the group made at least one selection in time to be considered for data analyses. Thus, after they had been assigned to a group, timed-out participants and their respective group were only excluded from further analyses if the timeout occurred in the first round. Otherwise, the rounds observed so far were used for analyses and the round before timing out was recorded as the final round.

2.2.1 Effects of drop-out on power

For our analysis of link selection, deviating from the incentivized choice, and majority convergence drop out and convergence only affect the number of observations per individual (and not higher levels). It should, therefore, not affect statistical power substantively. Group level analyses of assortment were also conducted across dropped out and non-dropped out groups, so that their statistical power should be unaffected by dropout. Finally, group level survival analyses account for drop out through censoring and power relies mainly on the number of (convergence or drop out) events. Thus, we are confident that convergence and dropout generally did not substantively affect the statistical power of our analyses.

2.2.2 Dropout speed and probability

Using a Cox-proportional Hazards model (see Supplementary Table 6), we find that link-determination conditions did not differ overall with regard to their probability of dropping out (see Supplementary Figure 5), $\chi^2(2) = 0.153$, $p = 0.926$, but did so as a function of link direction, $\chi^2(2) = 6.759$, $p = 0.034$. The effect was limited to outgoing links, Wald $\chi^2(3) = 8.05$, $p = 0.045$ [incoming links: Wald $\chi^2(3) = 2.19$, $p = 0.534$]. For outgoing links, we find that individuals who selected who to send their choice to were more likely to drop out, and dropped out faster, than if links were changing randomly, $b = 1.09$, $HR = 2.98$, $p = 0.060$, but not compered to static networks, $b = 0.62$, $HR = 1.85$, $p = 0.300$. This could indicate that this condition was more effortful and several participants decided to stop playing. It could also hint at technical issues. This would, however, be surprising since there is no apparent reason why technical issues should be selective to a single condition. Participants, in turn, might have experienced this condition as especially tedious, since they have little control over their observations, leading them to drop out more likely.

Using a logistic model, we did not find evidence for effects of our experimental factors on the probability of dropout (see Supplementary Table 1), except for a marginal interaction of link determination and link direction, Wald-$\chi^2(2) = 5.172$, $p = 0.075$ that appears driven by increased dropout for player selected links when links were outgoing, relative to randomly changing links $b = 0.92$, $OR = 2.51$, $p = 0.231$, although this relatively large effect cannot reliably be distinguished from zero. This interaction is in line with the results from the corresponding Cox-model on the speed of dropout. Overall, the model reflects minor differences in dropout between conditions. Even if this may render our performance results less convincing, it is unlikely to have affected our results concerning individual strategies.

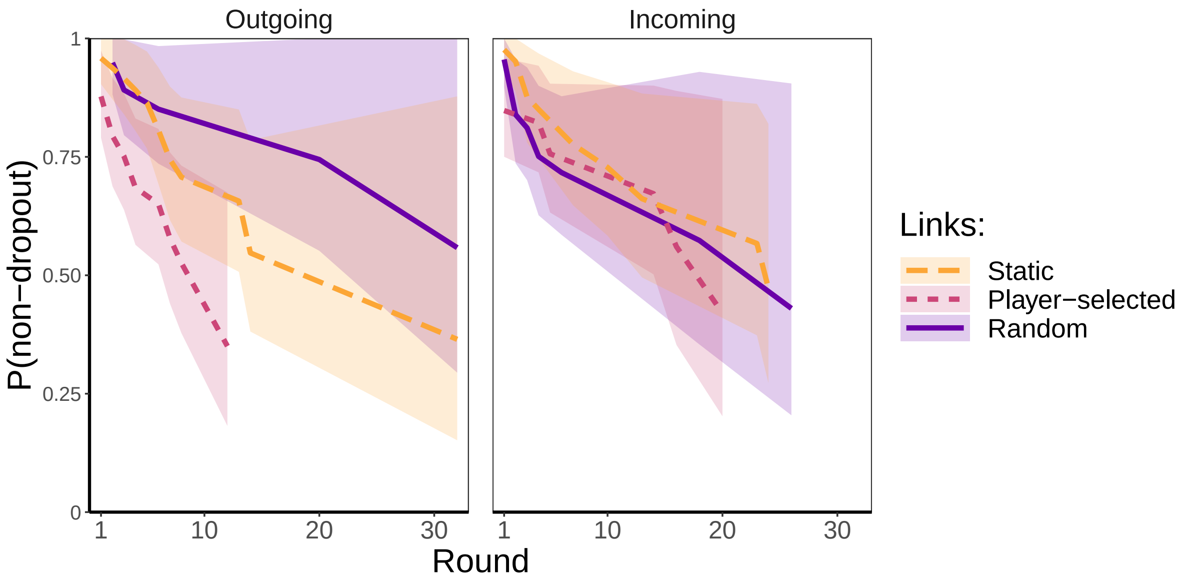

Supplementary Figure 5. Differences in dropout speed Survival plot of dropout as a function of link determination. Lines correspond to the probability of non-dropout in a given round. Shaded regions correspond to 95% confidence intervals.

Supplementary Table 1. Logistic models of the probability of dropout. All categorical predictors are on the network level and were effect coded. Effects are on the logit-scale, standard errors in parentheses.

	Dependent variable:	
	p(network dropout)	
	(1)	(2)
Constant	-1.055^{*} (0.146)	-1.165^{*} (0.167)
Link determination: Randomly changing		-0.092 (0.231)
Link determination: Player-selected links		-0.093 (0.254)
Link direction: Outgoing		0.150 (0.167)
Majority present		0.003 (0.167)
Randomly changing links × Link direction: Outgoing		-0.420^{+} (0.231)
Player-selected links × Link direction: Outgoing		0.502^{*} (0.254)
Randomly changing links × Majority present		0.156 (0.231)
Player-selected links × Majority present		0.202 (0.254)
Link direction: Outgoing × Majority present		-0.087 (0.167)
Randomly changing links × Link direction: Outgoing × Majority present		0.134 (0.231)
Player-selected links × Link direction: Outgoing × Majority present		-0.284 (0.254)
AIC	1537.09	609.78
Tjur's-R^2	0.00	0.04
LR-χ^2	0.00	9.42
Note:		N =244. $^{+}p < 0.1$; $^{*}p < 0.05$.

2.3 Statistical models

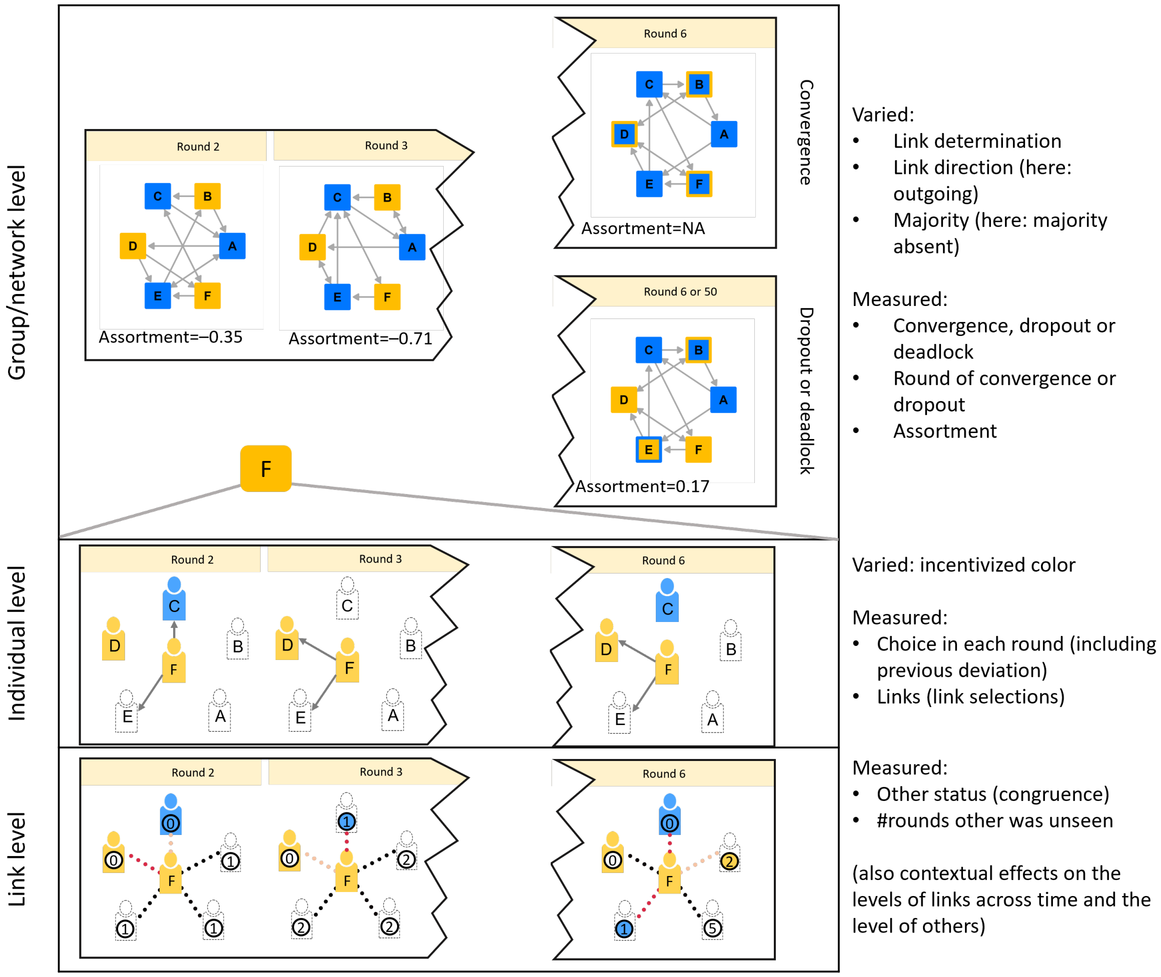

Supplementary Figure 6. Network characteristics and measured variables on different levels. On the network level (top) networks change and vary in their assortment. They either converge when all individuals agree on one color, drop out, when one individual leaves the study, or end up in deadlock, when they fail to agree within 50 rounds. Colored outlines correspond to the incentivized color. The lower 2 panels assume the perspective of individual F. On the individual level (middle) individuals make color choices and send their choice to other players (outgoing) or to observe them (incoming). On this level we measure individual choices and links. Individual F illustrates how links may change. In the outgoing condition individuals may observe any number of others between 0 and 5. The possible links (bottom) change their status over time with observation (see also Figure 1). On this level we measure how long others were unseen and what their last seen choice was. On all levels, fill colors correspond to color choices. Solid lines correspond to actual links, while dotted lines correspond to potential links between player-other pairs.

Here, we describe the models used in our analyses. They include the logistic mixed models to analyze link selection, choice behavior, and majority consensus in the final round, as well as survival models for convergence performance (and drop-out) and logistic models as robustness checks. We relied on mixed models whenever we had to account for non-independence of observations. In all mixed models, we simplified the random effects structure by removing random effects that could not be estimated.

All analyses were conducted in R-4.1.2[3]. Generalized mixed effects models were estimated using the function `glmer` in `lme4`[4]. We omitted random effects that could not be estimated based on the available data. Cox-proportional hazards models were estimated using `survival`[5].

We specified theoretically plausible models and tested the relevance of the corresponding predictors and their interactions. To test main effects and interactions we used the R-package `car`[6]. For fixed effects in logistic models (including logistic mixed models) we provide Wald-χ^2 statistics and their corresponding p-values[7]. These tests allow to test the relevance of variables in logistic models[8], including predictors with more than 2 levels[9], similar to omnibus-tests in an ANOVA framework. For survival models we provide likelihood ratio-based χ^2 tests.

Power considerations Our sample size was determined to detect small mean differences in an ANOVA framework (compare to pre-registration). In the context of random effects models, power mainly depends on the highest level in the hierarchy[10]. relatedly, all else being equal, within designs have more statistical power than between designs[11]. Because of this and because the estimation of power for more complex models is not straightforward without pre-existing data (but see[12, 13]), we limited our considerations to the network level. In addition to this, we provide models estimated with Bayesian techniques which provide more readily interpretable estimates of parameter uncertainty (see next paragraph).

Bayesian estimation to provide robustness We also estimated our main models using Bayesian techniques as robustness checks (Supplementary Tables 3, 5, 7, and 10). They provide estimates of our variables *given the data*. The 95% credibility intervals around each estimate indicate where the posterior estimate of an effect is expected to lie. They can be interpreted as uncertainty about the effects and provide a more straightforward indicator of whether an effect is likely (close to) zero or just unreliable due to limited amounts of data or noise (i.e., if the credibility interval includes zero and is relatively narrow, we can conclude that a given effect is likely negligible, otherwise the estimated effect may be inconclusive). In the supplementary results below we provide model tables and a brief discussion about each outcome variable next to each model.

Bayesian models were estimated using the package `brms`[14], *post hoc* tests were conducted using `emmeans`[15]. We provide median estimates and posterior density intervals, as provided by the software packages used. We ran all models for 4 chains with 4000 iterations and burn in of 2000 iterations to reduce auto-correlations. We used weakly informative priors ($b \sim \mathcal{N}(0,1)$) for estimates of main effects and interactions to shrink our estimates towards zero. For the intercept and random-effects parameters we used the default half-*t* distributions in `brms`. For correlations we used the default LKJ-priors.

2.3.1 Link selection

Our logistic mixed models of link selection predicted the probability of observing each possible link selection y as a function of features of the potential link (see also Figure 1b in main text and Supplementary Figure 6 lower panel), (1) including *observation status* of the other players (3-level factor if they agreed, or disagreed with the player's choice, or were not observed yet), (2) the number of rounds a player was unseen, and, on the group level, (3) the direction of links. The models are limited to the player-selected link condition (see Figure 1a and Figure 2 lower panel). If individuals had selected links randomly, in each round each link has a probability of $P(y = 1) = 0.4$, because 2 of 5 links were selected in each round. To control for non-independence we included random intercepts for each player-other pair and each other, as well as random slopes for the number of rounds unseen on the pair level. There was *a priori* no variation on the individual and network level, because the number, and hence the probability, of links for each individual and network was fixed. Therefore, we omitted random effects on these levels. However, pairs or others could vary in their probability of being selected (their popularity) beyond our measured factors, so that we included random effects on their levels.

We also tested the influence of round number (Supplementary Table 2 model 1) but did not find an effect and dropped it from further models. Beyond that, we assessed the robustness of our results by testing models with higher level contextual effects (models 3 to 5). This allows to investigate if the results can be explained by differences between pairs and others across all rounds, rather than the immediate features[16, 17]. Therefore, we centered the indicators of other status and number of rounds unseen on the pair mean (across rounds). To control for contextual effects we centered the pair means on the averages of the respective other and included both. These potential link means provide information about the average proportion of time that a link was in a given status (in terms of agreement and time unseen), whereas other means provide information on the popularity and agreement of an individual. Beyond that, we also tested the impact of available link types in each round, to exclude statistical artifacts due to different base rates (e.g., if the option to connect to agreeing others had been rarer).

2.3.2 Choice behavior

We estimated the probability $P(a \neq a_{\text{incentive}})_{ti}$ that a player did not select their incentivized color in a round as a function of characteristics of the round, including round number, whether an individuals already deviated in the previous round, the proportion of observed others deviating from the incentivized color (centered on 50% and scaled to steps of 10%), the number of others observed (centered on 2 other players), and the interaction of proportion deviating and number observed (see main text and Supplementary Table 4 model 2). The model includes a random intercept for players, random effects for round, and the proportion of others deviating. A network-level intercept $\sigma_{Network}$ and random effects for the number of other players were omitted in order to decrease model complexity and because they did not improve model fit.

Moreover, we tested contextual effects on the individual level and the level of networks, including our experimental factors (network-level; see Supplementary Table 4 model 3). We provide a model with round number only as reference (Supplementary Table 4 model 1).

2.3.3 Majority consensus

As pre-registered, we also estimated the probability that players i selected blue $P(a = 1|u)_i$ in the final round (with choice $a = 1$ corresponding to blue). In a logistic model we distinguish a network level and an individual level. On the *network level* we

introduced our experimental factors, including an indicator variable for whether a majority of yellow $X_j(n_{yellow} > n_{blue})$ or blue $X_j(n_{blue} > n_{yellow})$ was present, with a reference of absent majority $X_j(n_{yellow} = n_{blue})$, their interactions. We also entered the measured number of the final round. On the *individual level* we entered an indicator variable for the manipulated preference $x_{ij}(a_{incentive=1})$. To control for non-independence of observations we introduced a random intercept u_j for the network and allowed the effect of player incentive β_{5j} to vary by network.

The final round was defined as the last round for which we observed data. Therefore, all networks entered the analyses (including dropped out and converged networks). On the one hand, this procedure is conservative, because the majority exerted less influence in all non-converged networks (including dropped-out networks), on the other hand it retains statistical power by including all available information about the influence of the majority.

2.3.4 Survival models of convergence and drop out

Survival analyses of convergence and drop out estimated the differences in the hazard of converging or dropping out as a function of our experimental factors by using Cox proportional hazard models, like in previous network experiments[18,19]. These analyses naturally accounted for drop out like these previous network experiments did: Networks with drop out were considered as right-censored observations, which means that they were not considered at risk from the time of dropout. This censoring may have affected the power to detect smaller effects. Therefore, we interpret null findings with caution and under consideration of their effect size.

2.4 Measures

2.4.1 Assortment

Assortment in each round was calculated using the established assortativity coefficient by Newman[20], as implemented in the package `igraph`[21]. Assortativity r_a is based on the difference between the proportion of links e_{ii} that connect individuals with equal types i and the expected proportion of links with equal types $\sum_i a_i b_i$, normalized by the proportion of possible equal links beyond expectation $1 - \sum_i a_i b_i$.

$$r_a = \frac{\sum_i e_{ii} - \sum_i a_i b_i}{1 - \sum_i a_i b_i} \text{ where } a_i = \sum_j e_{ij}; b_i = \sum_i e_{ij}$$

It is larger if individuals of the same type are more likely connected to each other than expected from the number of links each type has. In our case, equal types were either be individuals who made the same choice in a given round (choice-based assortment) or who had the same incentive (incentive-based assortment).

2.4.2 Social value orientation

We also collected participants social value orientation[22], but do not discuss this measure further because analyses did not provide conclusive results.

3 Link selection

3.1 Individual link selection strategies

Robustness to contextual effects Overall, the pattern of results was unchanged when additional higher-level contextual effects, as well as the availability of agreeing and disagreeing others were included (Supplementary Table 2). We did not find evidence for an effect of round number (Model 1). Thus, it is omitted from other models and not further reported in the main text. (Note, that "round" is different from the "number of rounds" that the other in a pair was unseen, for which we do find a clear effect.) A main effect of others' *observation status*, Wald-$\chi^2(2) = 119.032$, $p < 0.001$, indicates that individuals are sensitive to whether they observed another player, and whether the other players agreed or disagreed with their own choice. Increased link probability with increasing *number of rounds unobserved* indicates that individuals prefer to select others who they currently have no information about, Wald-$\chi^2(1) = 85.165$, $p < 0.001$ (Supplementary Figure 7). The effects of observation status vary with number of rounds unseen and link direction, as indicated by 2-way interactions of status with number of rounds unseen, Wald-$\chi^2(2) = 40.010$, $p < 0.001$, status with link direction, Wald-$\chi^2(2) = 49.745$, $p < 0.001$, and number of rounds unseen with link direction, Wald-$\chi^2(1) = 75.562$, $p < 0.001$, and their 3-way interaction, Wald-$\chi^2(2) = 22.564$, $p < 0.001$. Therefore, we discuss simple effects for both link directions separately.

When individuals selected outgoing links, link-selection probabilities differed by *observation status*, $\chi^2(2) = 112.83$, $p < 0.001$, the *number of rounds unseen*, $\chi^2(1) = 19.53$, $p < 0.001$, and their interaction, $\chi^2(2) = 48.31$, $p < 0.001$ (Supplementary Figure 7). Individuals avoided sending their choice information to others agreeing with their last color choice, compared with both those they did not observe yet, $b = -0.99$, $OR = 0.37$, $Z = -6.64$, $p < 0.001$, and disagreeing others, $b = -0.72$, $OR = 0.48$, $Z = -9.74$, $p < 0.001$, with no difference between unobserved and disagreeing others, $b = 0.26$, $OR = 1.30$, $Z = 1.78$, $p = 0.210$. The link-selection probability increased with *number of rounds unobserved* for agreeing, $b = 0.26$, $OR = 1.29$, $Z = 6.36$, $p < 0.001$, but not for disagreeing, $b = -0.03$, $OR = 0.97$, $Z = -0.65$, $p = 0.513$, and marginally decreased for not yet observed others, $b = -0.13$, $OR = 0.88$, $Z = -2.00$, $p = 0.046$. Beyond the results reported above, this could indicate that individuals became increasingly less likely to send to those who were not observed for a large number of rounds. After not observing an other individual for 4 or more rounds, others' status no longer affected the probability that the player selected them, $\chi^2(2) = 0.02$, $p = 0.989$. Thus, individuals avoided sending to agreeing others, relying on memory for others' choices of the past 3 rounds.

When individuals selected incoming links, link-selection probabilities differed by *observation status*, $\chi^2(2) = 140.23$, $p < 0.001$, and *number of rounds unseen*, $\chi^2(1) = 94.65$, $p < 0.001$. In contrast to the results reported above, their interaction was not statistically significant, $\chi^2(2) = 2.36$, $p = 0.308$ (Supplementary Figure 7). Like those who selected outgoing links, they also avoided observing others agreeing with their last color choice, as compared with others whom they had not observed yet, $b = -1.55$, $OR = 0.21$, $Z = -11.36$, $p < 0.001$, and disagreeing others, $b = -0.37$, $OR = 0.69$, $Z = -5.83$, $p < 0.001$, with a preference for unobserved over disagreeing others, $b = 1.17$, $OR = 3.23$, $Z = 8.57$, $p < 0.001$. The link-selection probability increased with *number of rounds unobserved* for agreeing, $b = 0.49$, $OR = 1.63$, $Z = 12.42$, $p < 0.001$, disagreeing, $b = 0.45$, $OR = 1.57$, $Z = 11.09$, $p < 0.001$, and unobserved others, $b = 0.41$, $OR = 1.50$, $Z = 5.91$, $p < 0.001$, alike. Diverging from the analyses reported above, others' status still affected the probability that the player selected them after not observing an other individual for 4 or more rounds, $\chi^2(2) = 42.90$, $p < 0.001$. Instead others' status stopped reliably affecting the probability after 8 rounds, $\chi^2(2) = 4.94$, $p = 0.085$. Thus, individuals avoid observing agreeing others, relying on memory for others' choices of the last 7 rounds, which, however, happened rather rarely. This reflects uncertainty about the precise number of past rounds used in link selection. This further supports the finding that participants have a memory for those already observed, and that they avoid those relatively to others they did not yet observe.

Overall, these results still support that individuals sought out information about others they had not seen for a while and avoided selecting links to agreeing others, remembering others' previous choices for a few rounds. In sum, individuals preferentially avoid linking with those who agree with them, and thus avoid creating assortment of choices.

Diverging from the model reported in the main text, when we directly compare *link direction* conditions, individuals in the outgoing links condition were more likely to select currently disagreeing others (number of rounds unobserved = 0), $b = 1.02$, $OR = 2.79$, $Z = 11.05$, $p < 0.001$, but were also more likely to select currently agreeing others, $b = 0.34$, $OR = 1.41$, $Z = 3.57$, $p < 0.001$. They also were equally likely to observe others who had not been observed yet, $b = 0.19$, $OR = 1.21$, $Z = 1.07$, $p = 0.284$, compared to the incoming links condition. Thus, individuals more likely sent their choice to others who they had information about (outgoing links). Still, this was stronger for disagreeing than agreeing others, potentially reflecting relatively increased avoidance of disagreeing others more when they selected their observations (incoming links).

Bayesian estimation Supplementary Table 3 illustrates that our conclusions concerning link-selection are robust to alternative estimation techniques. Credibility intervals support that effects of observed links are affected by other status (agreeing, never seen), the number of rounds unseen, and their interaction with link direction. The pattern of results still holds true when controlling for contextual effects and experimental factors (model 3). Odds ratios not including 1 indicate that we could

Supplementary Table 2. Probability of a link between player and other Link probabilities in the player-selected condition as a function of number of rounds unobserved, other's status, link direction and their interactions. Model (2) is reported in the main text. Models (3) to (5) also include contextual effects on the other- and player-other level. Effects for "Other" pertain to others across rounds and individuals, effects for "Pair" pertain to player-other pairs. All other effects reflect differences in a pair in a round. All categorical predictors were effect coded. "Pair" effects were other-centered. Effects are on the logit-scale, standard errors in parentheses.

	Dependent variable:				
	p(Link between player and other)				
	(1)	(2)	(3)	(4)	(5)
Constant	−0.426* (0.023)	−0.635* (0.038)	0.556* (0.117)	0.486 (0.285)	0.139 (0.292)
Round number	−0.0001 (0.002)				
Network level					
Link direction: Outgoing		0.056 (0.037)	−0.160 (0.117)	−0.336 (0.284)	−0.189 (0.288)
Link level					
Other agreed		−0.538* (0.038)		−0.542* (0.040)	−0.793* (0.077)
Other never seen		0.293* (0.057)		0.429* (0.059)	0.672* (0.075)
Number of rounds unseen		0.282* (0.027)		0.297* (0.025)	0.242* (0.026)
Number of rounds unseen × Other agreed		0.137* (0.022)		0.105* (0.020)	0.132* (0.022)
Number of rounds unseen × Other never seen		−0.090* (0.032)		−0.044 (0.030)	−0.103* (0.034)
Cross level: network × link					
Link direction: Outgoing × Other agreed		−0.162* (0.038)		−0.064 (0.040)	−0.089* (0.040)
Link direction: Outgoing × Other never seen		−0.013 (0.058)		−0.192* (0.060)	−0.164* (0.061)
Link direction: Outgoing × Number of rounds unseen		−0.137* (0.027)		−0.197* (0.024)	−0.209* (0.024)
Link direction: Outgoing × Number of rounds unseen × Other agreed		0.055* (0.022)		0.081* (0.021)	0.091* (0.021)
Link direction: Outgoing × Number of rounds unseen × Other never seen		−0.007 (0.032)		−0.052 (0.030)	−0.060* (0.030)
Robustness controls: contextual effects other					
Mean proportion agreement			−1.465* (0.147)	−0.887 (0.458)	−0.472 (0.466)
Mean not yet seen			−0.226 (0.126)	−0.495 (0.312)	−0.198 (0.320)
Mean rounds unseen			−0.141* (0.056)	−0.302 (0.233)	−0.325 (0.237)
Mean proportion agreement × Mean rounds unseen				−0.217 (0.402)	−0.115 (0.408)
Mean not yet seen × Mean rounds unseen				−0.298 (0.258)	−0.549* (0.263)
Mean not yet seen × Link direction: Outgoing			0.260* (0.126)	−0.150 (0.312)	−0.166 (0.316)
Mean rounds unseen × Link direction: Outgoing			0.165* (0.056)	0.285 (0.231)	0.198 (0.235)
Mean proportion agreement × Mean rounds unseen × Link direction: Outgoing				−0.191 (0.401)	−0.048 (0.407)
Mean not yet seen × Mean rounds unseen × Link direction: Outgoing				0.804* (0.258)	0.836* (0.262)
Mean proportion agreement × Link direction: Outgoing			−0.224 (0.147)	0.118 (0.458)	−0.065 (0.464)
Robustness controls: contextual effects pair					
Proportion rounds agreed				−0.277* (0.082)	−0.404* (0.085)
Mean not yet seen				−0.804* (0.165)	−1.067* (0.173)
Mean rounds without link				−0.290* (0.032)	−0.283* (0.033)
Proportion rounds agreed × Mean rounds without link				−0.013 (0.113)	0.008 (0.115)
Mean not yet seen ×Mean rounds without link				−0.004 (0.148)	0.108 (0.150)
Link direction: Outgoing × Mean rounds without link				0.503* (0.032)	0.492* (0.032)
Link direction: Outgoing × Proportion rounds agreed				−0.104 (0.082)	−0.098 (0.083)
Link direction: Outgoing × Mean not yet seen				0.783* (0.164)	0.914* (0.167)
Link direction: Outgoing × Proportion rounds agreed × Mean rounds without link				0.046 (0.113)	0.092 (0.115)
Link direction: Outgoing × Mean not yet seen × Mean rounds without link				0.079 (0.148)	0.025 (0.149)
Robustness controls: individual availability					
Number agreeing available					0.219* (0.027)
Number disagreeing available					0.110* (0.027)
Other agreed × Number agreeing available					−0.107* (0.034)
Other never seen × Number agreeing available					0.087* (0.043)
Other agreed × Number disagreeing available					0.123* (0.035)
Other never seen × Number disagreeing available					0.032 (0.043)
σ_{Other}	0.075	0.08			
σ_{Pair}	0.453	0.599	0.409	0.581	0.596
$\sigma_{Pair\text{: Number rounds unseen}}$		0.49		0.373	0.367
$\rho_{Pair\text{: Intercept,Rounds unseen}}$		-0.687		-0.948	-0.936
AIC	22675.45	21986.52	22519.40	21254.41	21102.45
Nakagawa's-R^2	0.00	0.15	0.02	0.18	0.19
$LR\text{-}\chi^2$		712.93***	166.05***	1483.05***	1647.00***
Note:	N = 2370. $^{+}$ p < 0.1; * p < 0.05; ** p < 0.01; *** p < 0.001.				

Supplementary Table 3. Probability of a link between player and other (Bayesian estimation) Link probability between player and other in the player-selected condition as a function of number of rounds unobserved, other's status, link direction and their interactions. Model (2) is reported in the main text. Models (3) to (5) also include contextual effects on the other- and player-other level. Effects for "Other" pertain to others across rounds and individuals, effects for "Pair" pertain to player-other pairs. All other effects reflect differences in a pair in a round. All categorical predictors were effect coded. Contextual effects were centered on the next higher level. "Pair" effects were other-centered. Effects are on the logit-scale, 95% credibility intervals in parentheses.

	Dependent variable:	
	p(Link between player and other)	
	(1)	(2)
Intercept	−0.67 [−0.74, −0.59]	0.06 [−0.42, 0.55]
Network level		
Link direction: Outgoing	0.08 [0.01, 0.16]	−0.18 [−0.65, 0.28]
Link level		
Other agreed	−0.53 [−0.61, −0.46]	−0.78 [−0.94, −0.63]
Other never seen	0.28 [0.16, 0.39]	0.65 [0.51, 0.80]
Number of rounds unseen	0.32 [0.26, 0.37]	0.27 [0.21, 0.32]
Number of rounds unseen × Other agreed	0.13 [0.09, 0.18]	0.13 [0.09, 0.18]
Number of rounds unseen × Other never seen	−0.08 [−0.15, −0.02]	−0.10 [−0.17, −0.03]
Cross-level: network × link		
Link direction: Outgoing × Other agreed	−0.17 [−0.25, −0.09]	−0.09 [−0.18, −0.01]
Link direction: Outgoing × Other never seen	0.01 [−0.10, 0.13]	−0.15 [−0.27, −0.03]
Link direction: Outgoing × Number of rounds unseen	−0.17 [−0.22, −0.11]	−0.23 [−0.28, −0.18]
Link direction: Outgoing × Number of rounds unseen × Other agreed	0.06 [0.02, 0.11]	0.09 [0.05, 0.13]
Link direction: Outgoing × Number of rounds unseen × Other never seen	−0.02 [−0.09, 0.04]	−0.06 [−0.13, 0.00]
Robustness controls: contextual effects other		
Mean proportion agreement		−0.38 [−1.14, 0.38]
Mean not yet seen		−0.15 [−0.76, 0.43]
Mean rounds unseen		−0.26 [−0.65, 0.14]
Mean not yet seen × Mean rounds unseen		−0.58 [−1.08, −0.09]
Mean proportion agreement × Mean rounds unseen		−0.22 [−0.89, 0.47]
Mean not yet seen × Link direction: Outgoing		−0.14 [−0.74, 0.44]
Mean proportion agreement × Link direction: Outgoing		−0.08 [−0.83, 0.67]
Mean rounds unseen × Link direction: Outgoing		0.19 [−0.20, 0.57]
Mean not yet seen × Mean rounds unseen × Link direction: Outgoing		0.82 [0.33, 1.32]
Mean proportion agreement × Mean rounds unseen × Link direction: Outgoing		−0.01 [−0.68, 0.67]
Robustness controls: contextual effects pair		
Proportion rounds agreed		−0.40 [−0.57, −0.23]
Mean not yet seen		−1.05 [−1.40, −0.72]
Mean rounds without link		−0.28 [−0.35, −0.22]
Mean not yet seen × Mean rounds without link		0.07 [−0.24, 0.37]
Proportion rounds agreed × Mean rounds without link		0.01 [−0.22, 0.25]
Link direction: Outgoing × Mean not yet seen		0.90 [0.58, 1.23]
Link direction: Outgoing × Proportion rounds agreed		−0.09 [−0.26, 0.08]
Link direction: Outgoing × Mean rounds without link		0.49 [0.43, 0.56]
Link direction: Outgoing × Mean not yet seen × Mean rounds without link		0.05 [−0.25, 0.36]
Link direction: Outgoing × Proportion rounds agreed × Mean rounds without link		0.11 [−0.12, 0.34]
Robustness controls: individual availability		
Number agreeing available		0.22 [0.16, 0.27]
Number disagreeing available		0.11 [0.05, 0.16]
Other agreed × Number agreeing available		−0.11 [−0.18, −0.04]
Other never seen × Number agreeing available		0.09 [0.00, 0.17]
Other agreed × Number disagreeing available		0.12 [0.05, 0.19]
Other never seen × Number disagreeing available		0.04 [−0.05, 0.12]
σ_{Other}	0.09 [0.01, 0.19]	0.04 [0.00, 0.10]
σ_{Pair}	0.65 [0.57, 0.72]	0.64 [0.57, 0.72]
$\sigma_{Pair:\ Number\ rounds\ unseen}$	0.56 [0.49, 0.64]	0.42 [0.36, 0.48]
$\rho_{Pair:\ Intercept, Rounds\ unseen}$	−0.69 [−0.77, −0.59]	−0.92 [−0.96, −0.87]

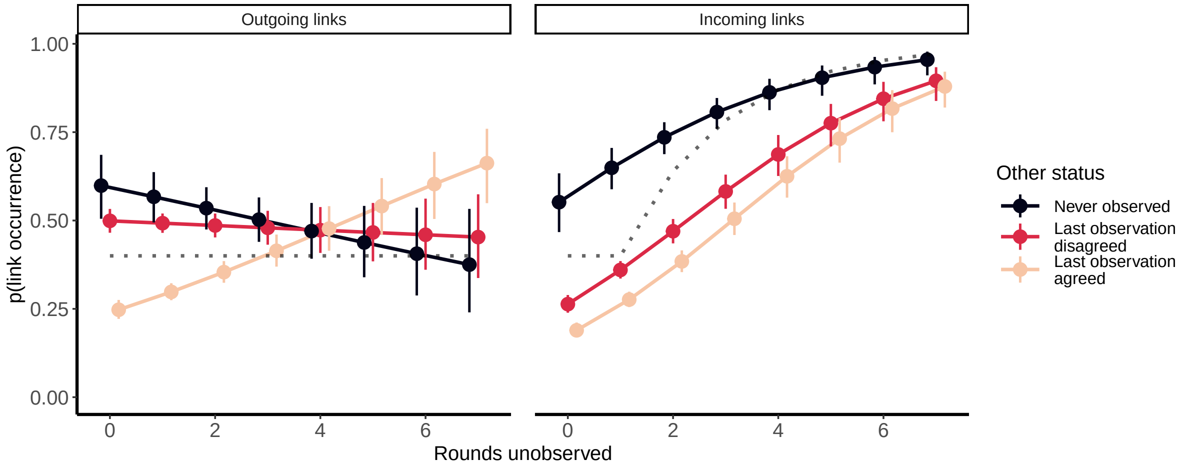

Supplementary Figure 7. Probability of a link between a player-other pair controlled for the availability. Colors indicate other's last seen choice and panels correspond to link direction. Zero rounds unobserved indicates that an other is observed during link selection (unshaded region). The dotted line indicates the probability of observing a link by chance (since 2 out of 5 targets are selected, this is a function of p = 0.4). Since 97.5% of all links were observed before an individual was unseen for 7 or more rounds, we truncated the plot at this value. Error bars correspond to 95% confidence intervals. (See also model 3 in Table 2).

estimate the direction of the effect reliably and narrower intervals indicate more certainty about the estimate given our data. Probing of the interaction of other status and link direction confirm that links to agreeing others were less likely both for outgoing links (agreeing vs. unseen: $b = -0.58$, $OR = 0.56$ $[0.47, 0.65]$; agreeing vs. disagreeing: $b = -0.73$, $OR = 0.48$ $[0.42, 0.55]$) and incoming links (agreeing vs. unseen: $b = -0.45$, $OR = 0.64$ $[0.54, 0.74]$; agreeing vs. disagreeing: $b = -0.34$, $OR = 0.71$ $[0.64, 0.79]$). Unseen others were estimated to be selected less likely than disagreeing others when links were outgoing ($b = -0.15$, $OR = 0.86$ $[0.73, 1.01]$), with no substantive difference when links were incoming (unseen / disagreeing: $b = 0.11$, $OR = 1.12$ $[0.94, 1.30]$).

In line with the idea of limited memory, differences between status decreased with the number of rounds unseen (after 4 rounds unseen all $1 < OR_{outgoing} < 1.25$ and all $1 < OR_{incoming} < 1.15$). We acknowledge that comparably large credibility intervals could indicate that differences for larger numbers of rounds unseen were estimated with less certainty and that memory effects were more persistent. However, this is unlikely, because typically human working memory is limited around that magnitude[23].

3.1.1 Link selection limited to immediate observations

The distribution of individuals' link selections based on others' currently visible color during their color choice corroborates the main result from our mixed logistic model (Supplementary Figure 8). A repeated measures ANOVA reveals that individuals across rounds were most likely to select links to others who they currently could not observe (including those not observed yet and those not observed for one round or more), followed by others who currently disagreed, and finally by those who agreed with their color choice, $F(2, 944) = 825.42$, $\eta^2_G = 0.636$, $p < 0.001$. Non-visible others were more likely to be selected when links were incoming, and disagreeing others were more likely to be selected when links were outgoing, $F(2, 944) = 10.05$, $\eta^2_G = 0.021$, $p < 0.001$. This further underscores that individuals avoid communicating with others who agree to avoid deadlocks. Additionally, that the tendency to select those who disagree is less pronounced when one observes others than if one sends their choice to others, may help individuals to avoid systematically creating the false impression of a majority against their incentive.

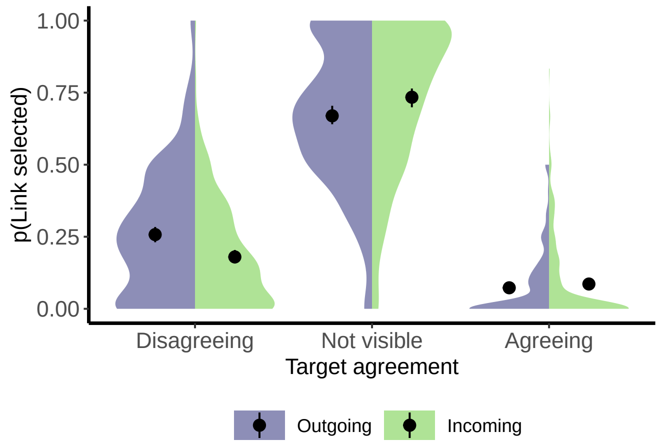

Supplementary Figure 8. Link selection probabilities across rounds. Distribution of individuals' link selection probabilities across rounds by observation status during the decision. Those who are currently unseen are selected most likely, followed by those who were disagreeing, and agreeing respectively.

3.2 Group Assortment

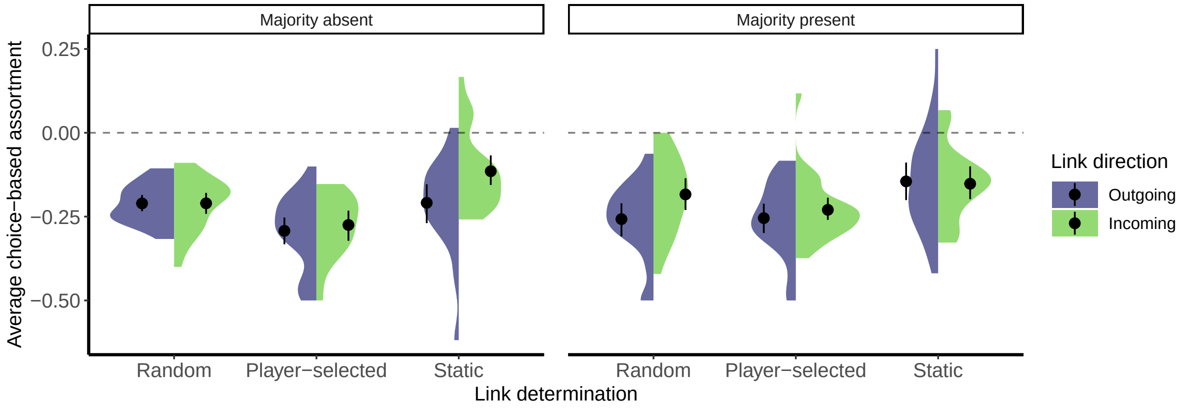

Supplementary Figure 9. Assortment based on players' choices (as reported in main text). Higher values indicate that players with the same choices were more likely connected. Assortment was largest in static networks absent majority. Player selected networks seemed to be lower in assortment, indicating stronger dis-assortment.

3-way interaction of link determination, link direction, and majority The 3-way interaction of link determination, link direction, and majority reported in the main text, $F(2,232) = 3.35$, $\eta^2_G = 0.028$, $p = 0.037$ shows that assortment is sometimes lower in player-selected than in randomly changing networks. Assortment in networks with player-selected links was also lower than in randomly changing networks with outgoing links and no majority, $b = -0.08$, $d = -0.73$, $t(232) = -2.38$, $p = 0.018$, and incoming links and a majority, $b = -0.08$, $d = -0.81$, $t(232) = -2.54$, $p = 0.012$, but not in networks with outgoing links and a majority, $b = -0.02$, $d = -0.15$, $t(232) = -0.45$, $p = 0.654$, and networks with incoming links and no majority, $b = -0.05$, $d = -0.48$, $t(232) = -1.38$, $p = 0.169$.

Incentive-based assortment In line with our pre-registration we also addressed assortment by individuals' preferences (or incentives), where higher values indicate that those who have the same incentive (rather than chose the same color, as shown above) are more likely to be connected by a link. As observed for choice-based assortment, incentive-based assortment is generally below zero indicating that no strong clustering, but rather dis-assortment, is observed. At odds with our pre-registred expectation, individuals' selection strategies did not systematically reflect in differences in clustering by their incentivized preferences, measured through average network level assortment based on preferences. We do not find effects of link determination, $F(2,260) = 2.27$, $\eta^2_G = 0.017$, $p = 0.106$, link direction, $F(1,260) = 2.36$, $\eta^2_G = 0.009$, $p = 0.126$, or their interaction, $F(2,260) = 0.89$, $\eta^2_G = 0.007$, $p = 0.410$ (see Figure 10).

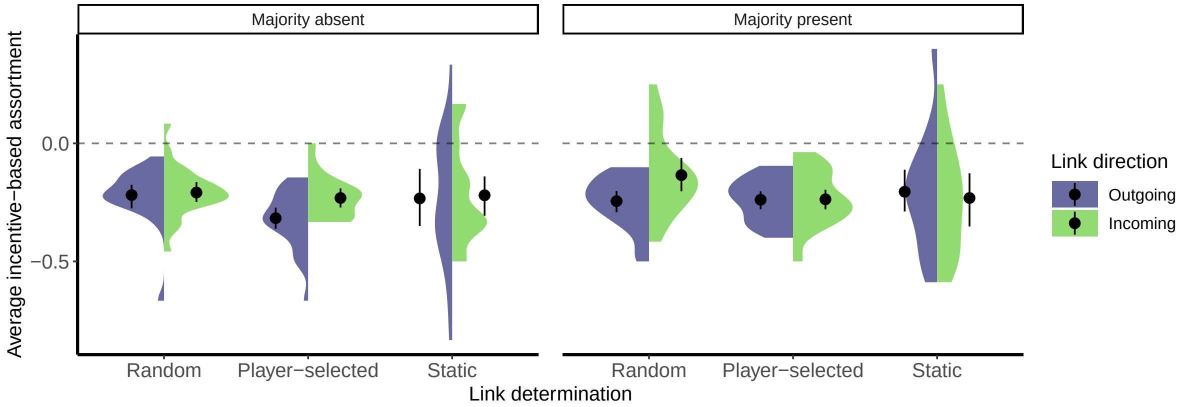

Supplementary Figure 10. Assortment based on players' incentivized color. Higher values indicate that players with the same incentive were more likely connected. Assortment varied most in static networks.

4 Choice behavior and agreement

4.1 Individual choice behavior

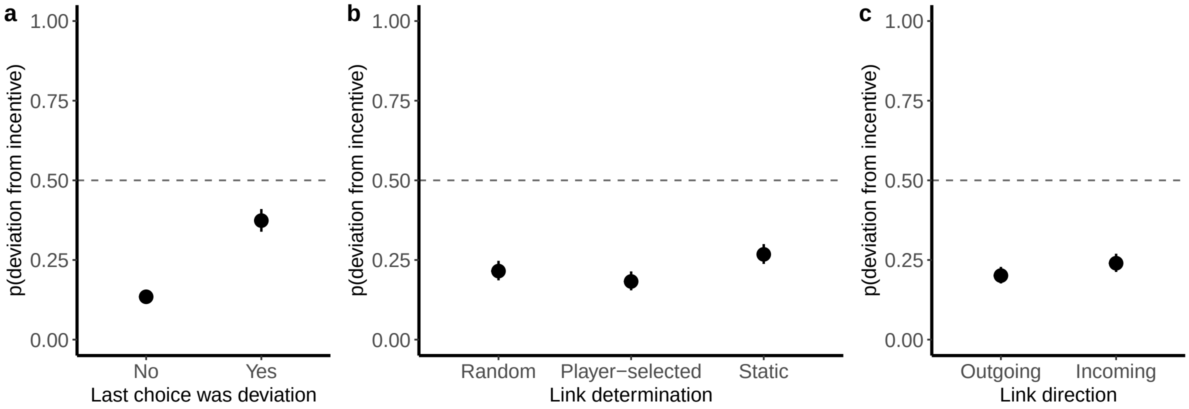

Supplementary Figure 11. Probability of deviating from the incentivized color by other predictors. Probability of a deviation from the incentivized color as a function of (a) whether the previous choice was already a deviation, (b) how links were determined, and (c) the direction of links. (a) Previous deviations make a subsequent deviation more likely, indicating inertia. (b) Deviations are more likely in static than in dynamic (randomly changing or player selected) networks. (c) Deviations are also more likely when links are incoming rather than outgoing.

Here we show that the above results about deviating from one's incentive are robust to the inclusion of experimental factors and aggregate indicators for deviations and observations on the individual and network level (see Supplementary Figures 11 and Supplementary Table 4). Interestingly deviations from one's incentive were less likely in dynamic networks (see Supplementary Figure 11b), Wald-$\chi^2(2) = 22.065$, $p < 0.001$, for outgoing links (see Supplementary Figure 11c), Wald-$\chi^2(1) = 6.598$, $p = 0.010$, and, marginally, in the presence of a majority, Wald-$\chi^2(1) = 3.552$, $p = 0.059$. Specifically, people were less likely to deviate as compared to static networks when they selected their interaction partners, $b = -0.49$, $OR = 0.61$, $Z = -4.62$, $p < 0.001$, or when links changed randomly, $b = -0.29$, $OR = 0.75$, $Z = -2.80$, $p = 0.015$, whereas randomly changing and player selected links did not differ, $b = 0.20$, $OR = 1.23$, $Z = 1.85$, $p = 0.182$. Moreover, individuals who sent their choices were less likely to deviate, $b = -0.23$, $OR = 0.80$, $Z = -2.57$, $p = 0.010$. In dynamic environments individuals may be more reluctant to deviate from their preference since they could obtain additional information about their fellow players, whereas their information in static networks is limited to their interaction partners. When links are outgoing, individuals may stick to their incentivized choice longer in order to communicate it to others and potentially convince them, whereas they are more likely to switch in cases where they are able to seek out information themselves.

Supplementary Table 4. Probability of deviating from incentive. Logistic mixed models of the probability that a player deviates from their incentivized color as a function of their observations and experimental factors, as well as individual-level averages capturing contextual effects. Models (2) and main text and model (3) is referenced as well. All categorical predictors were effect coded. Individual-level effects were network-centered. Other centering is noted in table. Effects are on the logit-scale, standard errors in parentheses.

	Dependent variable:		
	p(deviate from incentivized color)		
	(1)	(2)	(3)
Constant	-1.331^{*} (0.061)	-1.952^{*} (0.063)	-2.063^{*} (0.073)
Choice in round			
Round number	0.031^{*} (0.009)	0.009 (0.006)	0.011 (0.006)
Previous deviation from incentive		1.345^{*} (0.062)	1.310^{*} (0.062)
Number others observed (center: 2 individuals)		0.108^{*} (0.042)	0.130^{*} (0.048)
Proportion incentive deviations observed (center: 0.5, scale: 0.1)		0.342^{*} (0.014)	0.330^{*} (0.014)
Number others observed × Proportion incentive deviations observed		0.147^{*} (0.012)	0.149^{*} (0.012)
Individual level			
Player incentive			-0.105 (0.083)
Average individual observations			-0.091 (0.106)
Average individual deviations observed			0.134^{*} (0.029)
Average individual observations × Average individual deviations observed			0.017 (0.054)
Network level			
Majority present			-0.085 (0.045)
Link direction: Outgoing			-0.113^{*} (0.044)
Link determination: Randomly changing links			-0.028 (0.062)
Link determination: Player-selected links			-0.232^{*} (0.064)
Network average of observed deviations			-0.132^{*} (0.060)
Network average of blue incentives			-0.347 (0.391)
Link direction: Outgoing × Link determination: Randomly changing links			0.031 (0.061)
Link direction: Outgoing × Link determination: Player-selected links			-0.051 (0.063)
Link direction: Outgoing × Majority present			0.004 (0.044)
Link determination: Randomly changing links × Majority present			-0.003 (0.061)
Link determination: Player-selected links × Majority present			-0.046 (0.063)
Link direction: Outgoing × Link determination: Randomly changing links × Majority present			0.102 (0.061)
Link direction: Outgoing × Link determination: Player-selected links × Majority present			-0.090 (0.064)
σ_{Player}	1.473	1.022	1.041
σ_{Round}	0.135	0.059	0.06
$\rho_{Player:Intercept,Round}$	-0.273	-0.115	-0.137
$\sigma_{Proportion\ deviating}$		0.211	0.214
$\rho_{Player:Intercept,Proportion}$		-0.483	-0.498
$\rho_{Player:Round,Proportion}$		0.084	0.096
AIC	13968.58	12129.83	12095.16
Nakagawa's-R^2	0.01	0.26	0.28
LR-χ^2	***	2189.02^{***}	2257.69^{***}
Note:			$N = 13284$ ($n_{ind} = 1464$). $^{*}p < 0.05$

Bayesian estimation Supplementary Table 5 illustrates that our conclusions concerning deviations from the incentivized option are robust to alternative estimation techniques. Credibility intervals support that deviations and the number of others observed are larger than zero with substantive certainty, also when controlling for contextual effects and experimental factors. Like in the other models, compared to static networks, deviations were less likely when people selected their interaction partners, $b = -0.50$, $OR = 0.61\,[0.47, 0.74]$, and when links changed randomly, $b = -0.29$, $OR = 0.75\,[0.59, 0.91]$, whereas we cannot be certain that randomly changing and player-selected links differ, $b = 0.21$, $OR = 1.23\,[0.98, 1.54]$. Moreover, individuals who sent their choices were less likely to deviate than when links were incoming (see Supplementary Table 5).

Supplementary Table 5. Probability of deviating from the incentivized color (Bayesian estimates). Logistic mixed models of the probability that a player deviates from their incentivized color as a function of their observations and experimental factors, as well as individual-level averages capturing contextual effects. All categorical predictors were effect coded. Individual-level effects were network-centered. Other centering is noted in table. Effects are on the logit-scale, 95% credibility intervals in brackets.

	Dependent variable:	
	p(deviate from incentivized color)	
	(1)	(2)
Intercept	−1.94 [−2.07, −1.82]	−2.06 [−2.22, −1.92]
Choice in round		
Round number	0.01 [0.00, 0.02]	0.01 [0.00, 0.03]
Previous deviation from incentive	1.31 [1.19, 1.43]	1.27 [1.15, 1.39]
Number others observed (center: 2 individuals)	0.11 [0.02, 0.19]	0.13 [0.03, 0.22]
Proportion incentive deviations observed (center: 0.5, scale: 0.1)	0.34 [0.32, 0.37]	0.33 [0.30, 0.36]
Number others observed × Proportion incentive deviations observed	0.15 [0.13, 0.17]	0.15 [0.13, 0.18]
Individual level		
Player incentive		−0.11 [−0.28, 0.07]
Average individual observations		−0.10 [−0.31, 0.11]
Average individual deviations observed		0.15 [0.09, 0.21]
Average individual observations × Average individual deviations observed		0.03 [−0.09, 0.14]
Network level		
Majority present		−0.08 [−0.17, 0.01]
Link direction: Outgoing		−0.11 [−0.20, −0.02]
Link determination: Randomly changing links		−0.03 [−0.16, 0.10]
Link determination: Player-selected links		−0.24 [−0.37, −0.11]
Network average of observed deviations		−0.12 [−0.24, 0.00]
Network average of blue incentives		−0.28 [−1.02, 0.45]
Link direction: Outgoing × Link determination: Randomly changing links		0.03 [−0.10, 0.15]
Link direction: Outgoing × Link determination: Player-selected links		−0.05 [−0.18, 0.08]
Link direction: Outgoing × Majority present		0.00 [−0.09, 0.10]
Link determination: Randomly changing links × Majority present		0.00 [−0.12, 0.13]
Link determination: Player-selected links × Majority present		−0.05 [−0.18, 0.07]
Link direction: Outgoing × Link determination: Randomly changing links × Majority present		0.11 [−0.02, 0.24]
Link direction: Outgoing × Link determination: Player-selected links × Majority present		−0.09 [−0.23, 0.04]
σ_{Player}	1.06 [0.91, 1.22]	1.11 [0.96, 1.27]
σ_{Round}	0.06 [0.05, 0.08]	0.07 [0.05, 0.08]
$\sigma_{Proportion\ deviating}$	0.22 [0.19, 0.25]	0.22 [0.19, 0.25]
$\rho_{Player:Intercept,Round}$	−0.13 [−0.35, 0.12]	−0.16 [−0.37, 0.07]
$\rho_{Player:Intercept,Proportion}$	−0.40 [−0.54, −0.24]	−0.40 [−0.54, −0.24]
$\rho_{Player:Round,Proportion}$	0.05 [−0.21, 0.30]	0.06 [−0.20, 0.31]

4.2 Consensus performance

Supplementary Table 6. Cox-proportional hazards models on speed and probability of convergence (1) and dropout (2). Model (1) is reported in the main text. All categorical predictors are on the network level and were effect coded. Effects are on the log-scale, standard errors in parentheses.

	Dependent variable:			
	p(Hazard of convergence)		p(Hazard of drop-out)	
	(1)		(2)	
Randomly changing links (ref. static links)	0.002	(0.111)	−0.076	(0.206)
Player-selected links (ref. static links)	0.264^{*}	(0.113)	0.079	(0.233)
Link direction: Outgoing (ref. incoming)	0.047	(0.079)	0.165	(0.149)
Majority present (ref. majority absent)	0.069	(0.079)	0.059	(0.149)
Link determination: Random × Link direction: Outgoing	−0.016	(0.110)	-0.446^{*}	(0.205)
Link determination: Player-selected × Link direction: Outgoing	0.058	(0.112)	0.491^{*}	(0.231)
Random × Majority present	0.032	(0.110)	0.199	(0.205)
Player-selected × Majority present	0.002	(0.112)	0.213	(0.231)
Link direction: Outgoing × Majority present	0.190^{*}	(0.079)	0.039	(0.150)
Random × Link direction: Outgoing × Majority present	0.039	(0.112)	0.161	(0.208)
Player-selected × Link direction: Outgoing × Majority present	0.184	(0.112)	−0.159	(0.231)
AIC	1537.09		609.78	
R^2	0.064		0.052	
Wald Test (df = 11)	18.100^{+}		11.810	
Note:			N =244. $^{+}$ p < 0.1; * p < 0.05	

4.2.1 Convergence probability and speed

Bayesian estimation Supplementary Table 7 illustrates that our conclusions concerning the convergence speed are robust to alternative estimation techniques. Estimated effects are similar to the model estimated with maximum-likelihood techniques and conclusions from crdibility intervals align with statistical significance.

Supplementary Table 7. Bayesian Cox-proportional hazards models on speed and probability of convergence (1) and dropout (2). All categorical predictors are on the network level and were effect coded. Effects are on the log-scale, 95% credibility intervals in brackets.

	Dependent variable:			
	p(Hazard of convergence)		p(Hazard of drop-out)	
	(1)		(2)	
Intercept	1.11	[0.85, 1.39]	−0.16	[−0.60, 0.28]
Randomly changing links	0.01	[−0.21, 0.22]	−0.09	[−0.50, 0.31]
Player-selected links	0.25	[0.02, 0.47]	0.10	[−0.39, 0.52]
Link direction: Outgoing	0.05	[−0.11, 0.20]	0.19	[−0.09, 0.51]
Majority present	0.05	[−0.10, 0.21]	0.06	[−0.23, 0.37]
Randomly changing links × Link direction: Outgoing	−0.02	[−0.23, 0.20]	−0.46	[−0.87, −0.06]
Player-selected links × Link direction: Outgoing	0.06	[−0.16, 0.28]	0.50	[0.07, 0.97]
Randomly changing links × Majority present	0.03	[−0.19, 0.25]	0.21	[−0.19, 0.61]
Player-selected links × Majority present	0.01	[−0.22, 0.23]	0.23	[−0.19, 0.70]
Link direction: Outgoing × Majority present	0.18	[0.03, 0.34]	0.05	[−0.26, 0.34]
Randomly changing links × Link direction: Outgoing × Majority present	0.07	[−0.15, 0.28]	0.16	[−0.24, 0.57]
Player-selected links × Link direction: Outgoing × Majority present	0.16	[−0.06, 0.38]	−0.16	[−0.63, 0.26]

Logistic models of consensus probability As pre-registered, we tested our results for robustness using a logistic GLM on the probability of convergence as compared to both drop-out and non-convergence within 50 rounds (see Supplementary Table 8). We do not find reliable evidence for effects of our experimental factors on the probability of convergence (link determination: Wald-$\chi^2(2) = 0.994$, $p = 0.608$, link direction: Wald-$\chi^2(1) = 0.309$, $p = 0.578$, majority: Wald-$\chi^2(1) = 0.000$, $p = 0.988$), or their interactions (link determination × link direction: Wald-$\chi^2(2) = 3.558$, $p = 0.169$, link determination × majority: Wald-$\chi^2(2) = 3.268$, $p = 0.195$, link direction × majority: Wald-$\chi^2(1) = 0.631$, $p = 0.427$, link determination × link direction × majority: Wald-$\chi^2(2) = 1.886$, $p = 0.389$). This indicates that *whether* networks' reach consensus is not reliably affected by link determination, direction, or the presence of a majority, but how quickly they agree is.

Supplementary Table 8. Logistic models of the probability of convergence. All categorical predictors are on the network level and were effect coded. Effects are on the logit-scale, standard errors in parentheses.

	Dependent variable:	
	p(Convergence)	
	(1)	(2)
Constant	0.793* (0.138)	0.877* (0.151)
Randomly changing links		0.164 (0.213)
Player-selected links		0.026 (0.219)
Link direction: Outgoing		−0.083 (0.151)
EMajority present		−0.002 (0.151)
Randomly changing links × Link direction: Outgoing		0.397+ (0.213)
Player-selected links × Link direction: Outgoing		−0.212 (0.219)
Randomly changing links × Majority present		−0.199 (0.213)
Player-selected links × Majority present		−0.168 (0.219)
Link direction: Outgoing × Majority present		0.119 (0.151)
Randomly changing links × Link direction: Outgoing × Majority present		0.050 (0.213)
Player-selected links × Link direction: Outgoing × Majority present		0.216 (0.219)
AIC	304.69	317.10
Tjur's-R^2	0.00	0.04
$LR\text{-}\chi^2$	0.00	9.59
Note:		N=244. $^+p < 0.1$; $^*p < 0.05$.

4.2.2 Majority consensus

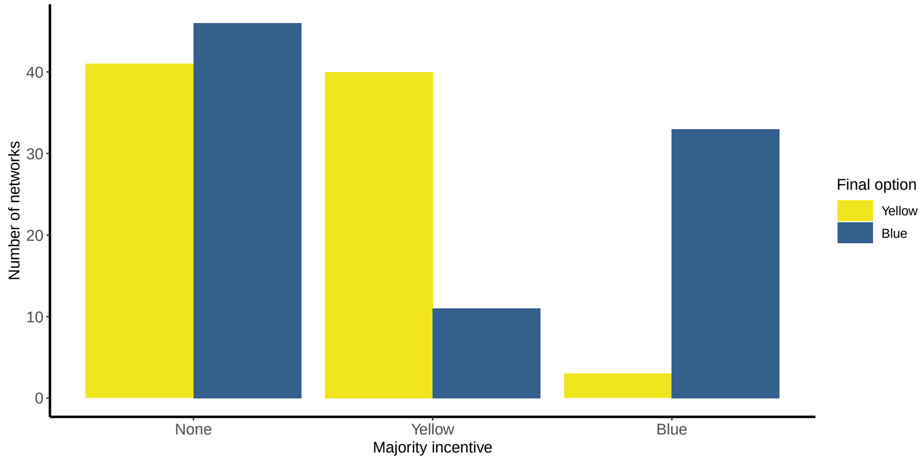

Supplementary Figure 12. Consensus on blue and yellow. Number of converged networks who agreed on blue and yellow by the respective majority (none, yellow, or blue).

In line with our pre-registered expectations, the color for which a majority was incentivized was the color the network typically agreed on (Supplementary Figure 12). However, in some static networks, blue won despite a yellow majority (see Supplementary Figures 13 and 14). In fact, the probabilities for a blue choice in the final round between a blue and a yellow majority were indistinguishable when links are static and incoming, $b = 0.00$, $OR = 1.00$, $p = 1.000$ (see Supplementary Figure 14). This shows that some static networks ended up being disadvantageous for the majority. Additional analyses indicate that stubborn individuals and deviations in round 1 may have been involved in these majority losses.

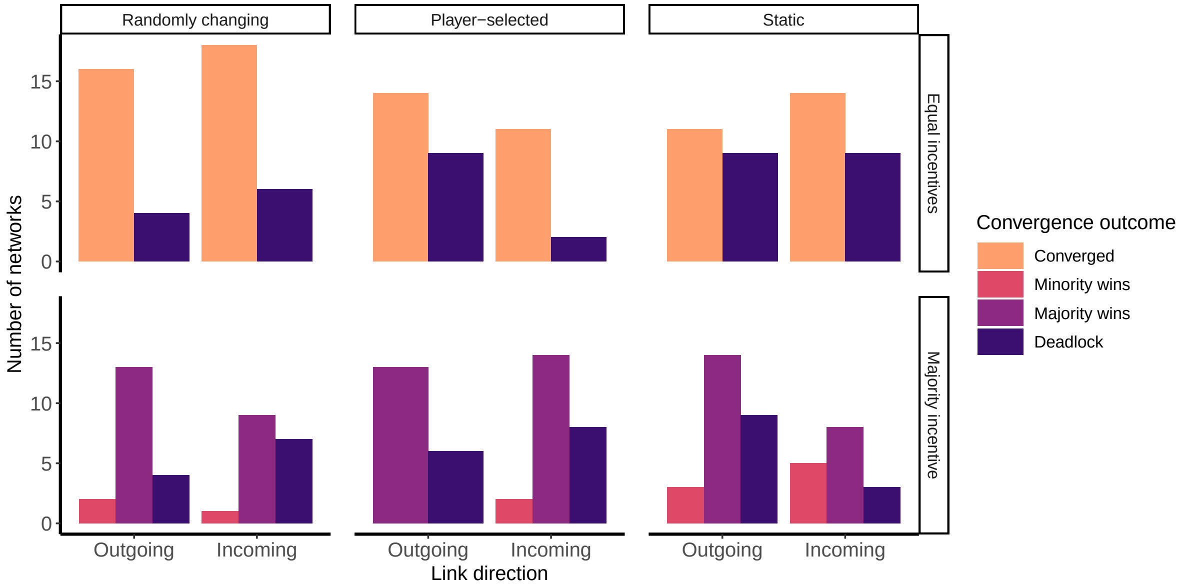

Supplementary Figure 13. Consensus outcomes across conditions. Consensus outcomes by link determination, link direction and majority condition. Deadlock was most likely in static networks. The probability that the network agreed on the color that was incentivized for a minority was also largest among static networks.

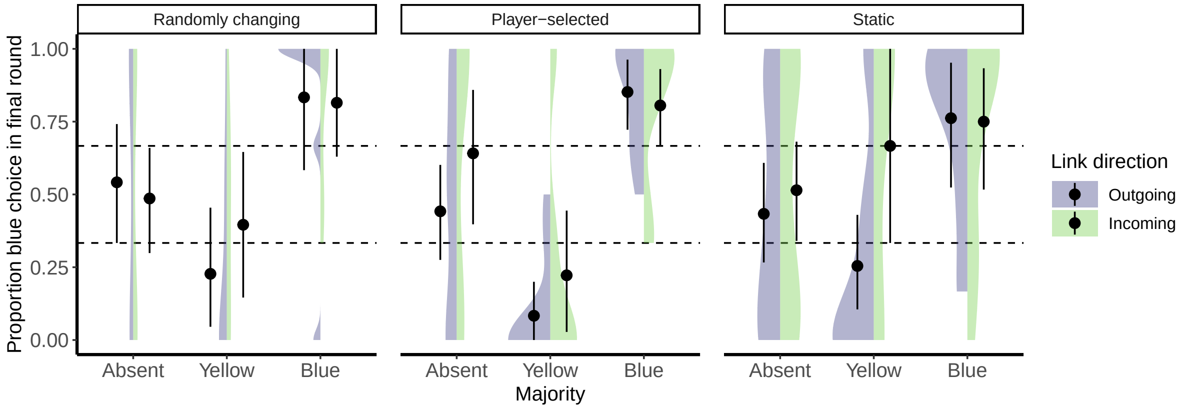

Supplementary Figure 14. Blue choices in the final round. Proportion of players' blue choices in the final round by link determination, link direction, and majority condition. Shaded areas indicate network-level distributions in data.

Supplementary Table 9. Individual probability of a blue choice in the final round. Shown as a function of experimental factors and incentive. Model (3) is reported in the main text. All categorical predictors were effect coded. Effects are on the logit-scale, standard errors in parentheses.

	Dependent variable:			
	p(Individual's final choice is blue)			
	(1)	(2)	(3)	(4)
Constant	0.109 (0.313)	0.813 (0.483)	1.148 (0.591)	1.148 (0.622)
Network level				
Final round number		−0.018 (0.034)	−0.025 (0.043)	−0.030 (0.044)
Yellow majority (ref. no majority)		-3.688^{*} (0.692)	-4.582^{*} (0.846)	-5.006^{*} (0.939)
Blue majority (ref. no majority)		4.007^{*} (0.692)	4.903^{*} (0.857)	5.287^{*} (0.953)
Randomly changing links		0.211 (0.532)	0.151 (0.650)	0.193 (0.677)
Player-selected links		−0.712 (0.530)	−0.866 (0.635)	−1.015 (0.661)
Link direction: Outgoing		-0.955^{*} (0.407)	-1.364^{*} (0.495)	-1.446^{*} (0.519)
Yellow majority × Randomly changing links		−0.363 (0.798)	−0.545 (0.963)	−0.218 (0.998)
Blue majority × Randomly changing links		0.554 (0.778)	0.741 (0.918)	0.403 (0.945)
Yellow majority × Player-selected links		-1.987^{*} (0.789)	-2.600^{*} (0.911)	-3.079^{*} (0.994)
Blue majority × Player-selected links		0.655 (0.766)	0.781 (0.884)	1.009 (0.933)
Yellow majority × Link direction: Outgoing		−1.095 (0.578)	-1.416^{*} (0.679)	-1.476^{*} (0.696)
Blue majority × Link direction: Outgoing		0.928 (0.569)	1.244 (0.679)	1.325 (0.716)
Randomly changing links × Link direction: Outgoing		0.664 (0.541)	0.927 (0.671)	1.014 (0.701)
Player-selected links × Link direction: Outgoing		0.010 (0.527)	0.062 (0.618)	−0.017 (0.649)
Yellow majority × Randomly changing links × Link direction: Outgoing		−0.188 (0.806)	−0.236 (0.976)	−0.338 (1.014)
Blue majority × Randomly changing links × Link direction: Outgoing		−0.450 (0.778)	−0.667 (0.925)	−0.765 (0.965)
Yellow majority × Player-selected links × Link direction: Outgoing		1.166 (0.809)	1.941^{*} (0.972)	1.936 (1.027)
Blue majority × Player-selected links × Link direction: Outgoing		0.174 (0.765)	0.210 (0.887)	0.237 (0.932)
Individual level				
Player incentive blue			1.045^{*} (0.155)	1.208^{*} (0.208)
Cross-level				
Yellow majority × Player incentive blue				0.072 (0.555)
Blue majority × Player incentive blue				0.191 (0.557)
Randomly changing links × Player incentive blue				−0.239 (0.228)
Player-selected links × Player incentive blue				0.440 (0.272)
Link direction: Outgoing × Player incentive blue				0.079 (0.221)
Yellow majority × Randomly changing links × Player incentive blue				0.049 (0.348)
Blue majority × Randomly changing links × Player incentive blue				−0.511 (0.335)
Yellow majority × Player-selected links × Player incentive blue				0.695 (0.523)
Blue majority × Player-selected links × Player incentive blue				−0.293 (0.355)
Yellow majority × Link direction: Outgoing × Player incentive blue				−0.016 (0.292)
Blue majority × Link direction: Outgoing × Player incentive blue				0.017 (0.266)
Randomly changing links × Link direction: Outgoing × Player incentive blue				0.077 (0.245)
Player-selected links × Link direction: Outgoing × Player incentive blue				0.007 (0.254)
Yellow majority × Randomly changing links × Link direction: Outgoing × Player incentive blue				−0.352 (0.345)
Blue majority × Randomly changing links × Link direction: Outgoing × Player incentive blue				−0.169 (0.347)
Yellow majority × Player-selected links × Link direction: Outgoing × Player incentive blue				0.153 (0.457)
Blue majority × Player-selected links × Link direction: Outgoing × Player incentive blue				−0.182 (0.341)
$\sigma_{Network}$	4.565	4.64	6.123	6.448
$\sigma_{Blue\ incentive}$			0.708	0.436
$\rho_{Network:\ Intercept, Blue\ incentive}$			0.169	0.647
AIC	1206.84	1158.80	1082.67	1096.39
Nakagawa's-R^2	0.00	0.31	0.34	0.37
$LR\text{-}\chi^2$		84.03^{***}	166.17^{***}	186.45^{***}
Note:	N=1464 ($n_{networks} = 244$). $^{+}p < 0.1$; $^{*}p < 0.05$; $^{**}p < 0.01$; $^{***}p < 0.001$.			

Supplementary Table 10. Individual probability of a blue choice in the final round (Bayesian fits). Shown as a function of experimental factors and incentive. All categorical predictors were effect coded. Effects are on the logit-scale, 95% credibility intervals in brackets.

	Dependent variable:	
	p(Individual's final choice is blue)	
	(1)	(2)
Intercept	0.58 [−0.54, 1.75]	0.59 [−0.68, 1.85]
Network level		
Final round number	−0.01 [−0.10, 0.07]	−0.01 [−0.10, 0.08]
Yellow majority (ref. no majority)	−2.01 [−3.07, −0.91]	−2.07 [−3.23, −0.89]
Blue majority (ref. no majority)	2.18 [1.08, 3.28]	2.28 [1.13, 3.45]
Randomly changing links	0.15 [−0.89, 1.22]	0.15 [−0.99, 1.29]
Player-selected links	−0.43 [−1.51, 0.63]	−0.46 [−1.57, 0.71]
Link direction: Outgoing	−0.83 [−1.68, −0.02]	−0.89 [−1.82, 0.01]
Yellow majority × Randomly changing links	−0.27 [−1.52, 1.00]	−0.24 [−1.51, 1.09]
Blue majority × Randomly changing links	0.30 [−1.04, 1.65]	0.25 [−1.05, 1.61]
Yellow majority × Player-selected links	−1.09 [−2.37, 0.19]	−1.08 [−2.42, 0.23]
Blue majority × Player-selected links	0.30 [−0.94, 1.56]	0.29 [−1.07, 1.61]
Yellow majority × Link direction: Outgoing	−0.66 [−1.73, 0.38]	−0.64 [−1.80, 0.50]
Blue majority × Link direction: Outgoing	0.46 [−0.63, 1.57]	0.41 [−0.77, 1.58]
Randomly changing links × Link direction: Outgoing	0.49 [−0.56, 1.56]	0.51 [−0.66, 1.66]
Player-selected links × Link direction: Outgoing	0.02 [−1.04, 1.09]	0.01 [−1.08, 1.14]
Yellow majority × Randomly changing links × Link direction: Outgoing	−0.06 [−1.33, 1.21]	−0.06 [−1.38, 1.27]
Blue majority × Randomly changing links × Link direction: Outgoing	−0.32 [−1.56, 0.95]	−0.35 [−1.67, 0.96]
Yellow majority × Player-selected links × Link direction: Outgoing	0.65 [−0.63, 1.93]	0.67 [−0.68, 1.99]
Blue majority × Player-selected links × Link direction: Outgoing	0.23 [−1.01, 1.49]	0.20 [−1.11, 1.54]
Individual level		
Player incentive blue	1.02 [0.75, 1.32]	1.27 [0.90, 1.68]
Cross level		
Yellow majority × Player incentive blue		0.15 [−0.41, 0.73]
Blue majority × Player incentive blue		0.10 [−0.42, 0.65]
Randomly changing links × Player incentive blue		−0.21 [−0.68, 0.26]
Player-selected links × Player incentive blue		0.35 [−0.12, 0.87]
Link direction: Outgoing × Player incentive blue		0.08 [−0.30, 0.46]
Yellow majority × Randomly changing links × Player incentive blue		0.18 [−0.48, 0.87]
Blue majority × Randomly changing links × Player incentive blue		−0.61 [−1.28, 0.04]
Yellow majority × Player-selected links × Player incentive blue		0.58 [−0.16, 1.35]
Blue majority × Player-selected links × Player incentive blue		−0.27 [−0.92, 0.39]
Yellow majority × Link direction: Outgoing × Player incentive blue		−0.02 [−0.60, 0.53]
Blue majority × Link direction: Outgoing × Player incentive blue		0.01 [−0.50, 0.52]
Randomly changing links × Link direction: Outgoing × Player incentive blue		0.09 [−0.40, 0.58]
Player-selected links × Link direction: Outgoing × Player incentive blue		−0.06 [−0.57, 0.44]
Yellow majority × Randomly changing links × Link direction: Outgoing × Player incentive blue		−0.30 [−0.97, 0.38]
Blue majority × Randomly changing links × Link direction: Outgoing × Player incentive blue		−0.18 [−0.85, 0.50]
Yellow majority × Player-selected links × Link direction: Outgoing × Player incentive blue		0.01 [−0.74, 0.75]
Blue majority × Player-selected links × Link direction: Outgoing × Player incentive blue		−0.15 [−0.80, 0.49]
$\sigma_{Network}$	5.91 [4.69, 7.40]	6.57 [5.21, 8.22]
$\sigma_{Blue\ incentive}$	0.57 [0.04, 1.13]	0.65 [0.05, 1.33]
$\rho_{Network:\ Intercept, Blue\ incentive}$	0.09 [−0.82, 0.88]	0.12 [−0.85, 0.91]

Supplementary Table 11. Logistic models of the probability that the majority wins. (1 and 2) Among all networks with majority present and (3 and 4) among all converged networks with a majority present. All categorical predictors are on the network level and were effect coded. Effects are on the log-scale, standard errors in parentheses.

	Majority convergence			
	Among all		Among converged networks	
	(1)	(2)	(3)	(4)
Constant	0.351^{+} (0.185)	0.359^{+} (0.190)	1.698^{*} (0.302)	4.432 (301.509)
Randomly changing links		0.086 (0.276)		−2.397 (301.509)
Player-selected links		0.196 (0.266)		5.824 (603.018)
Link direction: Outgoing		0.208 (0.190)		2.894 (301.509)
Randomly changing links × Link direction: Outgoing		0.120 (0.276)		−3.057 (301.509)
Player-selected links × Link direction: Outgoing		0.011 (0.266)		5.416 (603.018)
AIC	166.08	173.58	74.39	75.51
Tjur's-R^2	0.00	0.02	0.00	0.10
LR-χ^2	0.00	2.50	0.00	8.88
Note:				N=120 and $n_{conv} = 83$. $^{+}$p < 0.1; *p < 0.05.

Bayesian estimation Supplementary Table 10 illustrates that our conclusions concerning the majority color are robust to alternative estimation techniques. We find reliably differences between the final proportion yellow and blue across conditions with the exception of static networks with outgoing links ($p(blue|\text{yellow majority}) = 0.93[0.33, 1.00]$; all other $p(blue|\text{yellow majority}) < 0.5$). Together with the large credibility interval, this could indicate that the advantage could not be estimated properly because of some network structures that hindered the majority (compare to main text and see Section 5 below for examples).

Probability that the majority wins In line with our preregistration, we tested our results for robustness using a logistic GLM on majority consensus. We do not find evidence that our experimental factors affected whether the majority wins more likely than deadlock or or the minority (see Figure 13) across networks with a majority (link determination: Wald-$\chi^2(2) = 1.220$, $p = 0.543$, link direction: Wald-$\chi^2(1) = 1.204$, $p = 0.273$). When only considering converged networks, we find an effect of link determination Wald-$\chi^2(2) = 6.438$, $p = 0.040$, but not of link direction: Wald-$\chi^2(1) = 1.204$, $p = 0.273$.

Overall, detecting differences in convergence speed through survival models may be the more reliable method to detect how network determination affects performance, because qualitative differences in the final outcome are too small to be detected by our experimental design. Survival models may be the more powerful tool because they also detect qualitative differences between networks, that logistic models could not detect reliably.

5 Specific networks

Specific networks help illustrate possible reasons why some networks deviated against the majority color (see Supplementary Figures 15 to 17). Possible reasons include random errors away from the majority incentive, as well as highly stubborn individuals. Potentially, network structure played an additional role beyond that (see, e.g., Supplementary Figure 15).

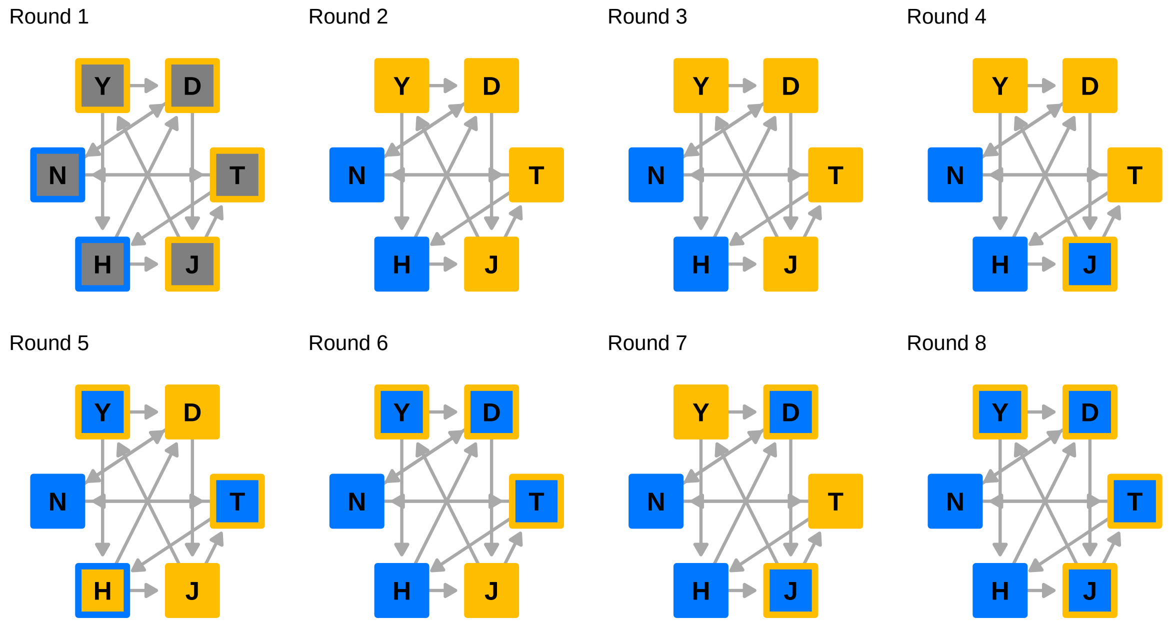

Supplementary Figure 15. Network example: stubborn minority on outgoing links. Borders correspond to individuals' incentivized colors and fill colors to the current color choice. This network agreed against the majority color. Visual inspection of choices indicates that player J initiated a change away from the majority option in round 4. This change cascaded through player Y to player D, who already observed 2 stubborn blue minority individuals N and H. Eventually the network converged on blue rather than yellow.

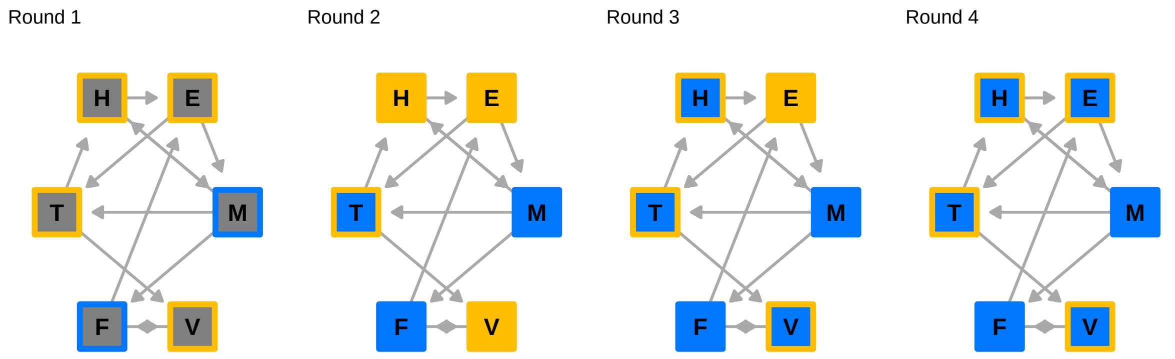

Supplementary Figure 16. Network example: initial deviation on incoming links. Borders correspond to individuals' incentivized colors and fill colors to the current color choice. Network and choices in a network that agreed against the majority color. Colored frames indicate the incentivized color, colored boxes individuals' choices. Visual inspection of choices indicates that player T deviated from their incentivized color in the first choice (Round 2). This may have led H and E to also deviate from their incentivized color, leading to consensus on the blue minority color.

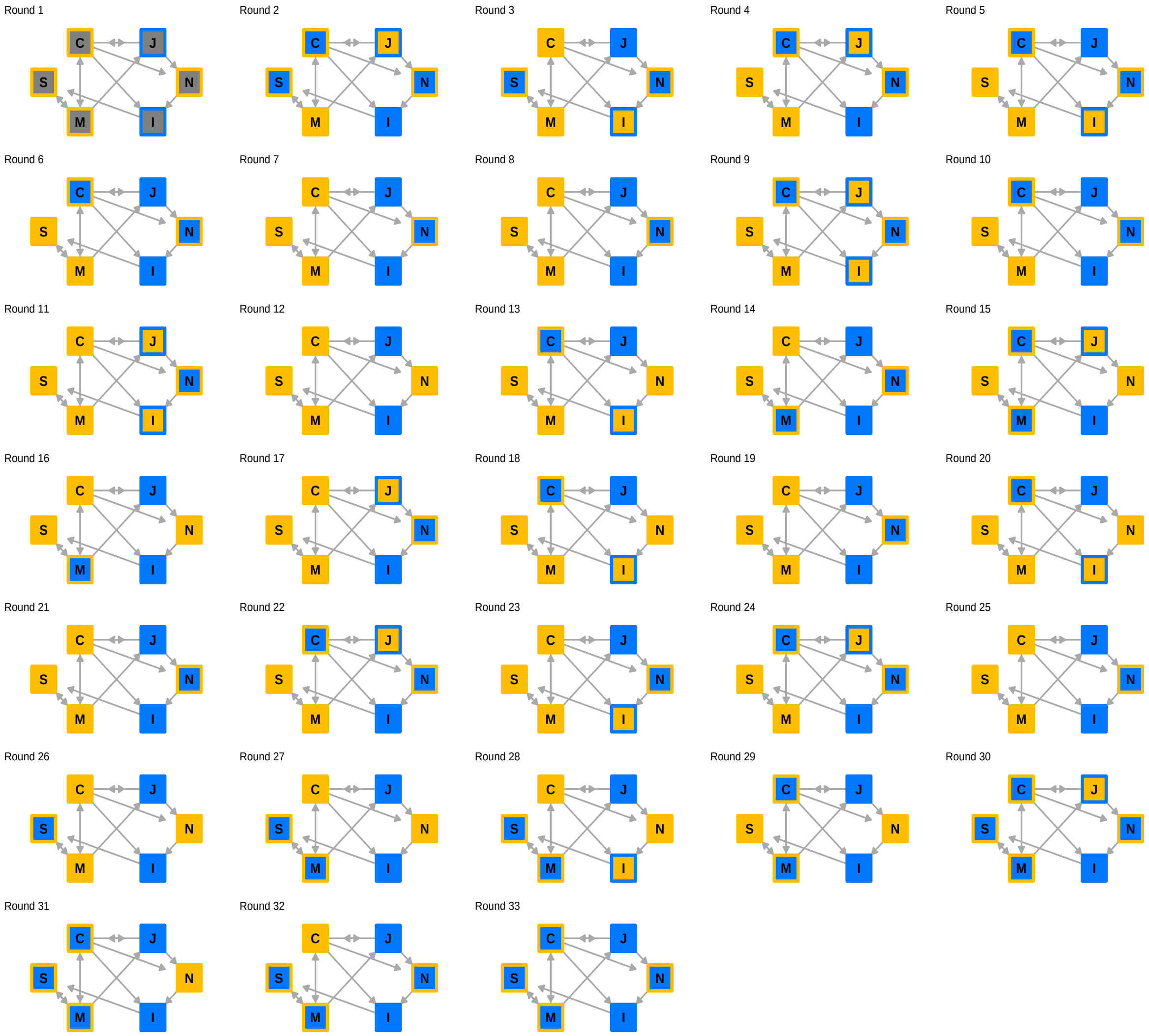

Supplementary Figure 17. Network example: multiple early deviations on incoming links. Borders correspond to individuals' incentivized colors and fill colors to the current color choice. Network and choices in a network that agreed against the majority color. Colored frames indicate the incentivized color, colored boxes individuals' choices. Visual inspection of choices indicates that deviations in the first rounds, followed by a relatively stubborn blue minority (Individuals I and J), which eventually wins.

6 Self-reported link-selection strategies

Supplementary Table 12. Classes of self reported strategies. κ indicates the inter-rater reliability. Numbers correspond to the count for each strategy class by rater 1 and 2 respectively, with the average proportion among classifiable statements the corresponding condition in parentheses.

Class	κ	n(class|rater 1, rater 2) (average proportion) by link direction: Outgoing		Incoming	
Self-reported behaviors					
Random	0.74	41,35	(0.19)	49,47	(0.27)
Select all others	0.64	40,34	(0.19)	89,67	(0.43)
Select invisible others	0.69	34,34	(0.17)	7, 8	(0.04)
Select agreeing others	0.65	21,16	(0.09)	4, 2	(0.02)
Select disagreeing others	0.75	88,80	(0.43)	34,32	(0.18)
Select others by (in-)stability	0.21	3, 1	(0.01)	30, 7	(0.10)
Select the same others	0.57	8, 9	(0.04)	16,16	(0.09)
Unspecified use of color	0.47	11,33	(0.11)	15,34	(0.14)
Not classifiable	0.61	69,63	(0.25)	65,70	(0.27)
Goals					
Gather or provide information	0.30	22,11	(0.08)	39,23	(0.17)
Convince others	0.53	42,26	(0.17)	2, 9	(0.03)
Reassure others	0.83	14,14	(0.07)	0, 1	(0.00)
Agree fast	0.59	18,19	(0.09)	8,15	(0.06)

To provide additional information about the strategies that people used to select their network links we classified participants' self-reports. For 511 individuals, we classified (a) the reported behaviors, as well as (b) any goals they reported. Behaviors included whether they selected others randomly, tried to select all others, selected invisible, agreeing or disagreeing others, or used color in any other way. Goals included whether participants explicitly stated that they wanted to convince or reassure others, or whether they adapted their behavior to agree fast (e.g., by switching their color). Thereby, each statement could belong to more than one class. We also classified whether a statement did not belong into any of those classes. All statements were classified by two raters. We only interpret those classes for which the inter-rater reliability Cohen's κ was at least 0.50.

Supplementary Table 12 shows the average proportion for each class among those statements across both raters. Although some individuals reported at least some random element in their strategy, the self-reports support that many people selected their network links strategically.

Thereby, the most frequent strategies vary by link direction. Generally, relatively many participants reported that they tried to select each other player at least once. This was reported more frequently when links were incoming. Individuals more frequently reported that they sent their color to invisible others and different others, but also similar others when links were outgoing.

Few individuals explicitly stated their goals, but when links were outgoing, individuals report both that they tried convince and reassure disagreeing others. Thus, reports from the incoming link condition suggest that individuals tried to get a complete picture of the other players. Conversely, reports from the outgoing link condition indicate that many individuals selected disagreeing others (likely with the goal of convincing them), while some tried to reassure others who already agreed. Thus, individuals selected their links in a strategic fashion, that is consistent with our behavioral results.

References

1. McElreath, R. *et al.* Beyond existence and aiming outside the laboratory: Estimating frequency-dependent and pay-off-biased social learning strategies. *Philos. Transactions Royal Soc. B: Biol. Sci.* **363**, 3515–3528, DOI: 10.1098/rstb.2008.0131 (2008).
2. Kunjar, S. *et al.* Link updating strategies influence consensus decisions as a function of the direction of communication. *Royal Soc. Open Sci.* **10**, 230215, DOI: 10.1098/rsos.230215 (2023).
3. R Core Team. *R: A Language and Environment for Statistical Computing*. R Foundation for Statistical Computing, Vienna, Austria (2021).
4. Bates, D., Maechler, M., Bolker, B. & Walker, S. *lme4: Linear Mixed-Effects Models using Eigen and S4* (2021). R package version 1.1-27.1.
5. Therneau, T. M. *survival: Survival Analysis* (2021). R package version 3.2-11.
6. Fox, J. & Weisberg, S. *An R Companion to Applied Regression* (Sage, Thousand Oaks CA, 2019), third edn.
7. Agresti, A. *Categorical data analysis* (Wiley-Interscience, Hoboken, NJ, 2002), 2. edn.
8. Jaeger, T. F. Categorical data analysis: Away from ANOVAs (transformation or not) and towards logit mixed models. *J. Mem. Lang.* **59**, 434–446, DOI: 10.1016/j.jml.2007.11.007 (2008).
9. Wald, A. Tests of statistical hypotheses concerning several parameters when the number of observations is large. *Transactions Am. Math. Soc.* **54**, 426–482 (1943).
10. Snijders, T. A. Power and sample size in multilevel modeling. *Encycl. Stat. Behav. Sci.* **3**, 1573 (2005).
11. Maxwell, S. E. & Delaney, H. D. *Designing experiments and analyzing data: a model comparison perspective* (Lawrence Erlbaum, Mahwah, NJ [u.a.], 2004), 2 edn.
12. Aguinis, H., Gottfredson, R. K. & Culpepper, S. A. Best-practice recommendations for estimating cross-level interaction effects using multilevel modeling. *J. Manag.* **39**, 1490–1528, DOI: 10.1177/0149206313478188 (2013).
13. Mathieu, J. E., Aguinis, H., Culpepper, S. A. & Chen, G. Understanding and estimating the power to detect cross-level interaction effects in multilevel modeling. *J. Appl. Psychol.* **97**, 951–966, DOI: 10.1037/a0028380 (2012).
14. Bürkner, P.-C. Bayesian item response modeling in R with brms and Stan. *J. Stat. Softw.* **100**, 1–54, DOI: 10.18637/jss.v100.i05 (2021).
15. Lenth, R. V. *emmeans: Estimated Marginal Means, aka Least-Squares Means* (2022). R package version 1.7.2.
16. Enders, C. K. & Tofighi, D. Centering predictor variables in cross-sectional multilevel models: a new look at an old issue. *Psychol. Methods* **12**, 121–138, DOI: 10.1037/1082-989X.12.2.121 (2007).
17. Snijders, T. A. & Bosker, R. J. *Multilevel analysis: An introduction to basic and advanced multilevel modeling* (Sage, 2011).
18. Gaisbauer, F., Strandburg-Peshkin, A. & Giese, H. Local majority-with-inertia rule can explain global consensus dynamics in a network coordination game. *Soc. Networks* **70**, 218–227, DOI: 10.1016/j.socnet.2022.01.013 (2022).
19. Giese, H., Gaisbauer, F., Gradwohl, N. & Strandburg-Peshkin, A. The role of position in consensus dynamics of polarizable networks. *Sci. Reports* **13**, 3972, DOI: 10.1038/s41598-023-30613-z (2023).
20. Newman, M. E. J. Assortative mixing in networks. *Phys. Rev. Lett.* **89**, 208701, DOI: 10.1103/PhysRevLett.89.208701 (2002).
21. Csardi, G. & Nepusz, T. The igraph software package for complex network research. *InterJournal* **Complex Systems**, 1695 (2006).
22. Murphy, R. O., Ackermann, K. A. & Handgraaf, M. Measuring social value orientation. *Judgm. Decis. Mak.* **6**, 771–781, DOI: 10.1017/S1930297500004204 (2011).
23. Cowan, N. The magical mystery four: How is working memory capacity limited, and why? *Curr. Dir. Psychol. Sci.* **19**, 51–57, DOI: 10.1177/0963721409359277 (2010).
